# *Pseudomonas aeruginosa* AES-1 Exhibits Increased Virulence Gene Expression during Chronic Infection of Cystic Fibrosis Lung

**DOI:** 10.1371/journal.pone.0024526

**Published:** 2011-09-15

**Authors:** Sharna Naughton, Dane Parker, Torsten Seemann, Torsten Thomas, Lynne Turnbull, Barbara Rose, Peter Bye, Stuart Cordwell, Cynthia Whitchurch, Jim Manos

**Affiliations:** 1 Department of Infectious Diseases and Immunology, University of Sydney, Sydney, Australia; 2 Department of Microbiology, Monash University, Melbourne, Australia; 3 Victorian Bioinformatics Consortium, Monash University, Melbourne, Australia; 4 School of Biotechnology and Biomolecular Sciences and Centre for Marine Bio-Innovation, University of New South Wales, Sydney, Australia; 5 The ithree institute, University of Technology, Sydney, Australia; 6 Department of Respiratory Medicine, Royal Prince Alfred Hospital, Sydney, Australia; 7 Sydney Medical School, University of Sydney, Sydney, Australia; 8 School of Molecular Biosciences, University of Sydney, Sydney, Australia; University of Birmingham, United Kingdom

## Abstract

*Pseudomonas aeruginosa,* the leading cause of morbidity and mortality in people with cystic fibrosis (CF), adapts for survival in the CF lung through both mutation and gene expression changes. Frequent clonal strains such as the Australian Epidemic Strain-1 (AES-1), have increased ability to establish infection in the CF lung and to superimpose and replace infrequent clonal strains. Little is known about the factors underpinning these properties. Analysis has been hampered by lack of expression array templates containing CF-strain specific genes. We sequenced the genome of an acute infection AES-1 isolate from a CF infant (AES-1R) and constructed a non-redundant micro-array (PANarray) comprising AES-1R and seven other sequenced *P. aeruginosa* genomes. The unclosed AES-1R genome comprised 6.254Mbp and contained 6957 putative genes, including 338 not found in the other seven genomes. The PANarray contained 12,543 gene probe spots; comprising 12,147 *P. aeruginosa* gene probes, 326 quality-control probes and 70 probes for non-*P. aeruginosa* genes, including phage and plant genes. We grew AES-1R and its isogenic pair AES-1M, taken from the same patient 10.5 years later and not eradicated in the intervening period, in our validated artificial sputum medium (ASMDM) and used the PANarray to compare gene expression of both in duplicate. 675 genes were differentially expressed between the isogenic pairs, including upregulation of alginate, biofilm, persistence genes and virulence-related genes such as dihydroorotase, uridylate kinase and cardiolipin synthase, in AES-1M. Non-PAO1 genes upregulated in AES-1M included pathogenesis-related (PAGI-5) genes present in strains PACS2 and PA7, and numerous phage genes. Elucidation of these genes' roles could lead to targeted treatment strategies for chronically infected CF patients.

## Introduction

In cystic fibrosis (CF) patients, chronic *Pseudomonas aeruginosa* infection and inflammation are associated with biofilm formation, a progressive decline in lung function and premature death [1], [2], [3]. Early infection of CF infants with *P. aeruginosa* is usually successfully managed by aggressive antibiotic therapy; however by adolescence most patients have chronic infection. Prominent persisters include the frequent clones Liverpool Epidemic Strain (LES), Manchester strain (c3719), and the Australian Epidemic Strain-1 (AES-1), which infects ca. 40% of adult CF patients in Sydney and Melbourne [4], [5]. While frequent clones are often more resistant to antibiotics than infrequent clones, they lack a distinctive antibiotic resistance profile [6], indicating that other factors are involved in their persistence.

*P. aeruginosa* undergo a number of genetic and expression changes that assist in their ability to survive in the CF lung and to evade detection and clearance by the immune system [7], [8]. Acute lung infection in CF patients is associated with expression of virulence determinants that are involved in establishment of infection in animal model systems [9], [10], [11], [12]. However, *P. aeruginosa* from chronically-infected CF patients usually lack some of these virulence determinants, suggesting that these genes are unnecessary for long-term maintenance of *P. aeruginosa* infection in vivo [13], [14], [15].

Genomic studies of two closely related *P. aeruginosa* strains collected from a CF patient 7.5 years apart [14] showed loss of function mutations in virulence genes required for O-antigen biosynthesis, Type III secretion (T3SS), twitching motility, exotoxin A regulation, multidrug efflux, osmotic balance, phenazine biosynthesis, quorum sensing, and iron acquisition. Chronic infection strains also possess particular characteristics, including large chromosomal inversions (LCI) [16], a specific Type IV pilin allele [17], exhibit enhanced biofilm dispersal properties [18] and a progressive loss of T3SS function over time [19]. In vivo conditions can also result in genomic changes to persistent strains and this is probably related to strain hypermutability [20], [21].

Persistence may also be related to changes in levels of expression of particular genes. Conversion to the mucoid phenotype, which is dependent on biofilm formation, has been associated with establishment of chronic infection [22]. Chronic growth also appears to activate new virulence strategies, including the catabolism of fatty acids such as phosphotidylcholine and prostaglandin, thus preventing their utilisation by the host [12]. A study of sequential isogenic *P. aeruginosa* isolates from three adults with chronic infection showed upregulation of anaerobic respiration, microaerobic respiration and the TCA cycle pathways [23], however there have been no studies comparing the gene expression changes between isogenic acute infection and chronic infection isolates. Additionally, the sequencing of the acute AES-1 isolate AES-1R and the use of an array encompassing eight *P. aeruginosa* genomes has allowed detection of novel genes not present in the PAO1 genome.

This study has used an artificial sputum media closely mimicking CF sputum (ASMDM) [24] and an array based on the genome sequence of AES-1R and other CF isolates (PANarray) to identify the gene expression changes in sequential early and chronic isogenic AES-1 isolates.

## Results

### Homology between AES-1 and other P. aeruginosa genomes

**Figure 1:**
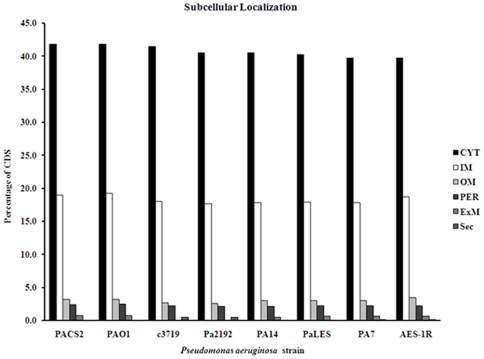
Subcellular localization of *P. aeruginosa* AES-1R genes with known functions. Gene products of known function were classified into one of six localization sites (cytoplasmic-CYT, inner membrane-IM, outer membrane-OM, exomembrane-ExM, periplasmic-PER, secreted-sec) for each of the eight *P. aeruginosa* genomes on the PANarray. AES-1R had a significantly lower cytoplasmic protein content (39.7%) compared to PACS2 (41.8%), PAO1 (41.8%) and c3719 (41.4%) (Pearson's χ^2^ test: p = 0.0014, p = 0.017 and p = 0.047, respectively).

In order to identify all coding sequences (CDS) in the eight *P. aeruginosa* genomes (AES-1, PAO1, PA7/PSPA7, PA14, PACS2, Pa_2192, Pae/PALES and c3719), BLAST analysis was performed on the AES-1R sequence, which produced 7672 clusters, of which 3962 represented CDS common to all strains, while between 54 and 338 CDS were unique to one of the eight genomes, based on an E-value of less than 10^−4^ (Table 1). The AES-1R genome comprised 6,254,604 bases, the second smallest genome on the PANarray after c3719. Overall 7199 putative CDS were detected, which after the elimination of 242 adjoining duplicates gave an adjusted total of 6957 CDS. This is higher than the average for the seven other PANarray genomes (5784) though the number might fall if the genome sequence is closed, as other genes may have been duplicated. The predicted subcellular location of CDS products (Fig. 1) showed that AES-1R has a significantly lower proportion of cytoplasmic proteins (39.7%) than the genomes of PACS2 (41.8%), PAO1 (41.8%) and c3719 (41.4%), (Pearson's χ^2^ test: p = 0.0014, p = 0.017 and p = 0.047, respectively,). In terms of cluster of orthologous groups (COG) function (not shown), a comparison of AES-1R with PAO1, PA14, Pa2192 and PA7 showed no significant differences in group size (p<0.05) with the exception of inorganic ion transport and metabolism, where AES-1R had a significantly greater number of CDS (388 against 291) compared to PA7 (p = 0.035).

**Table 1:** Genomic statistics of the *P. aeruginosa* genomes on the PANarray.

Bacterial strain	Size (Mbp)	Total CDS§	Unique CDS	% of genome
*P. aeruginosa* AES-1R	6.254	6957†	338	4.9%
*P. aeruginosa* Pa2192	6.826	5914	284	4.8%
*P. aeruginosa* PACS2	6.492	5676	44	0.8%
*P. aeruginosa* UCBPP-PA14	6.537	5896	183	3.1%
*P. aeruginosa* PAO1	6.264	5568	73	1.3%
*P. aeruginosa* c3719	6.146	5221	63	1.2%
*P. aeruginosa* PaLES	6.601	5925	193	3.3%
*P. aeruginosa* PSPA7	6.439	6286	54	0.9%

§ CDS  =  Coding sequences.

† Total adjusted for adjoining duplicates.

All genes were annotated based on BLAST analysis results, with genes showing an E-value of less than 10^−4^ designated as unique.

AES-1R had the highest number of unique CDS of the eight genomes (338), though as a percentage of total CDS, AES-1 was at the same level as *P. aeruginosa* Pa2192 (Table 1). The unique AES-1R genes coded mainly for hypothetical proteins, but also included several characterized proteins such as the SOS-response transcriptional repressor *lexA* (AES_7031), three Mu-like bacteriophages (AES_7010, 7011 and 7084), the bacteriophage P2 tail protein *gpl*, heme exporter protein *ccmA* and polyhydroxyalkanoate synthesis protein *phaF* (Table S1). Fully 67.4% of CDS in the region between AES_6966 and AES_7152 are unique to AES-1R (E value less than 10^∼4^ for all homologs), indicating that this 187 CDS region may be part of the AES-1R accessory genome. While most CDS in this region are hypothetical proteins, 16 have putative or probable phage functions.

### Genotyping and growth characteristics of CF strains in ASMDM

**Figure 2:**
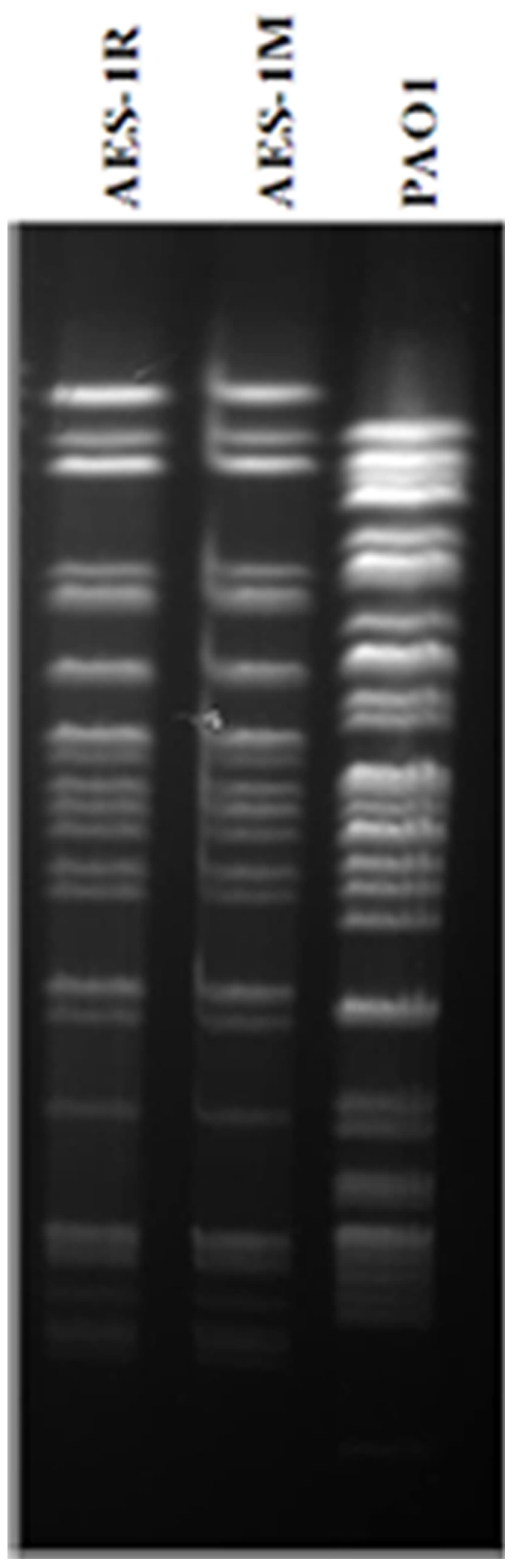
Genotyping of *P. aeruginosa* AES-1R, AES-1M and PAO1 by pulsed field gel electrophoresis. Bacterial cells embedded in agarose plugs were lysed using EC lysis buffer (Sigma-Aldrich Australia), and digested using restriction enzyme *SpeI* to generate a small number (ca. 15–40) of large DNA fragments. Band patterns were analysed using cluster analysis software (GelComparII™, Applied Maths, Belgium) and established criteria [55] (same genotype if less than two bands difference). The AES-1R and AES-1M genomes fall within these criteria.

**Figure 3:**
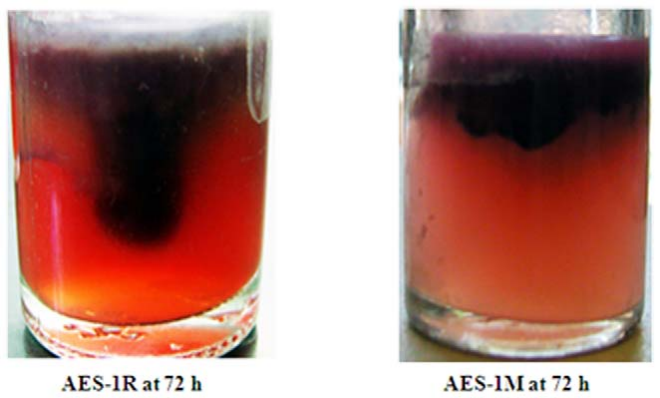
Phenotypic appearance of *P. aeruginosa* AES-1R and AES-1M in ASMDM at 72h. ASMDM was inoculated just under the surface with 50µl of a 1∶100 dilution of an overnight culture. Growing cells were identified by oxidation of triphenyl tetrazolium chloride (0.025% w/v final concentration) which stains cells a deep red colour.

Pulsed field gel electrophoresis (PFGE) of AES-1R and AES-1M showed no discernable band differences (Fig. 2) therefore they were classed as the same strain. The acute and chronic isolates both had a non-mucoid phenotype on horse blood agar (HBA) and Mueller-Hinton agar (MHA). This is unusual since most chronic isolates are mucoid or revertants from mucoid phenotypes [25], [26]. AES-1R and AES-1M may qualify as small colony variants (SCV) due to their small colony size of 2–5 mm after 48 hours, and the upregulation of the polysaccharide genes *pelC* and *pelE,* part of the *pelABCDEF* in AES-1M may be contributing to this morphotype (see Discussion) [27]. As shown in Fig. 3, there is a difference in the growth patterns of AES-1R and AES-1M. AES-1R grew with a thickened pellicle and projections into the media while AES-1M had smaller projections at 72h. When incubated for up to 96h, the growth of AES-1M resembled that of AES-1R (not shown), indicating that it is slower growing during chronic infection. The growth characteristics of AES-1M resembled those of *P. aeruginosa* UCBPP-PA14 in ASMDM [24], which also grew more slowly with smaller anaerobic projections.

### Overall changes in expression in AES-1 over time

In all, 675 genes were differentially-expressed between the frequent clone isolates AES-1R and AES-1M at p<0.05 (Table S2) including 365 upregulated and 310 downregulated in the chronic AES-1M. Five hundred and twenty five of these genes were differentially expressed ≥2fold, while 74 (11.2%) of all differentially expressed genes did not have homologues in the PAO1 genome sequence (Table S3). These genes would not have been detectable on the Affymetrix PAO1 array. They included 10 phage genes and one integrase gene, eight of which were upregulated (average upregulation 3.7fold), two upregulated pathogenesis-related proteins from *P. aeruginosa* gene island-5 (PAGI-5): PaerPA_01000873 (3.8fold) and PSPA7_4490 (5.8fold), the enzyme uridylate kinase (6.4fold), and the downregulated proteins polyketide synthase (−4.3fold) and luminal binding protein (−3.4fold).

### Gene expression differences between acute and chronic infection AES-1 isolates

Against expectations that chronic infection leads to a downregulation of virulence-related genes, a number of genes with virulence functions were upregulated in chronic AES-1M compared to its acute infection counterpart (Table 2). These included alcohol dehydrogenase *adhA,* (5.5fold), the T3SS regulator *pscD* (3.2fold), uroporphyrinogen decarboxylase *hemE* (16-fold), peptidyl-prolyl cis-trans isomerase *ppiA* (2.5-fold), alginate-associated genes *algD,E,F,8* and *amr*Z (1.9 to 10.7fold), dihydroorotase *pyrC* (12.1fold), uridylate kinase *pyrH* (6.4fold), the extracellular polysaccharide genes *pelC* (5.6fold) and *pelE* (3.7fold) part of the *pelABCDEF* operon, phospholipase N (*plcN*) (3.2fold) and cardiolipin synthase *cls* (7.0fold). Genes with putative or probable virulence roles upregulated in chronic AES-1M included a probable haloacid dehalogenase (21.6fold), threonine dehydratase *ilvA1* (9.7fold) xanthine phosphoribosyltransferase *xpt* (16.7fold) and polyhydroxyalkanoate synthesis protein *phaF* (6.0fold) (Table S2). The downregulation of homogentisate 1,2-dioxygenase *hmgA* (-7.5fold) is also virulence related since its downregulation de-represses pyomelanin production, which enhances persistence.

**Table 2:** Known virulence-related genes differentially expressed in *P. aeruginosa* AES-1M compared to AES-1R (p<0.05).

Gene name	FC§	Description	Role in *P. aeruginosa*
*hem*E	16.1	Uroporphyrinogen decarboxylase	Regulation of alginate secretion
*pyr*C	12.1	Dihydroorotase	Biofilm development, QS and virulence
*pyr*H	6.4	Uridylate kinase	Biofilm development, QS and virulence
*adh*A	5.5	Alcohol dehydrogenase	Required for biofilm development
*pel*B, *pel*E	5.6, 3.7	Extracellular polysaccharide	Competitive disruption of S. aureus biofilms
*cls*	7.0	Cardiolipin synthase	Increases membrane lipids and AB resistance
*psc*D	3.2	T3SS export protein	Regulator of T3SS - virulence
*plc*N	3.2	Phospholipase C precursor	Degrades phosphatidylcholine in lung surfactant
*alg*D,E,F,8,*amr*Z	1.9-10.7	Alginate biosynthesis	Biofilm development
*ppi*A	2.5	Peptidyl-prolyl cis-trans isomerase	ΔppiA-decreased growth- susceptible to AB*
*hmg*A	-7.2	Homogentisate 1-2-dioxygenase	Loss induces pyomelanin - greater persistence
*alg*C	-9.3	Phosphomannomutase	Loss = defective A band in lipopolysaccharide

§ FC  =  Fold change - indicates up or downregulated in AES-1M relative to AES-1R.

*AB  =  Antibiotic.

### Gene expression by quantitative PCR

**Figure 4:**
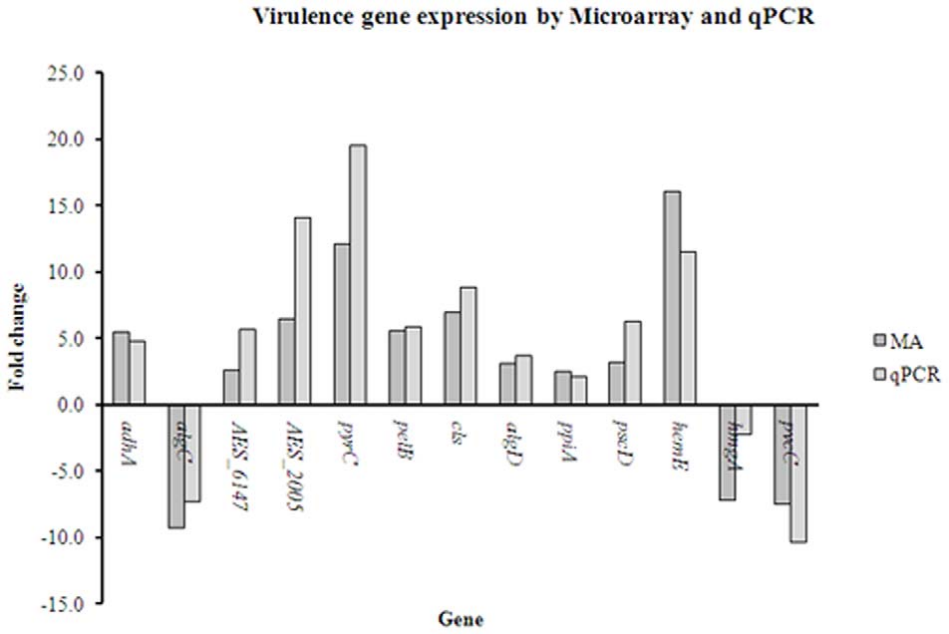
Differential expression of virulence-related genes by microarray and qPCR. Thirteen mainly virulence-related genes significantly differentially expressed by microarray were also quantified for expression by qRT-PCR. Five of these (*adhA*, *algC*, AES_6147, AES_2005 and *pyrC*) were quantified for qPCR using the same RNA sample as for microarray and eight (*pelB*, *cls*, *algD*. *ppiA*, *pscD*, *hemE*, *pvcC* and *hmgA*) were quantified using a different RNA sample to that for microarray, and gave an overall correlation coefficient R^2^ = 0.8053.

The average quantitative PCR ratios of the selected virulence-related (Table 2) and other genes correlated well with their microarray expression ratios (correlation coefficient: R^2^ = 0.8053 − Fig. 4). All genes showed either up- or downregulation consistent with the microarray results, despite eight of these being quantified using different RNA samples for array and qPCR.

## Discussion

The sequencing of the genome of the Australian epidemic strain AES-1 (isolate R) has provided the first opportunity to examine the similarities and differences between this frequent clone widespread in eastern Australia and other frequent clones such as PaLES and c3719. In terms of overall size, AES-1R (6.254 Mbp) falls between c3719 (6.146 Mbp) and PaLES (6.601 Mbp), and close to that of PAO1 (6.264 Mbp). As the c3719 and PaLES genomes are closed and the AES-1R genome is not, a direct comparison of CDS is not possible, however in terms of genes of known function there are significant differences between AES-1R and c3719. In subcellular localisation (Fig. 1) AES-1R has a significantly smaller proportion of cytoplasmic genes and a significantly greater proportion (3.4% against 2.7%) of outer membrane genes compared to c3719 (Pearson's χ^2^ test: p = 0.047 and p = 0.019, respectively,). With respect to unique CDS (Table 1), PaLES (3.3%) and c3719 (1.2%) have significantly smaller proportions compared to total CDS than does AES-1R (p<0.0001 for both comparisons). The proportion of unique CDS in AES-1R is similar to that for Pa2192 (p = 0.83). The 338 unique CDS (Table S1) are heavily concentrated between AES_6966 and AES_7152, thus there is a high likelihood that this 187-CDS region represents a portion of the AES-1R accessory genome. AES-1R contains at least one novel integrated prophage in this region, with 16 CDS having a known or putative phage function. Amongst these are two Mu-like prophage proteins (gp28 and gp29). Mu-like proteins have been identified in a number of pathogenic bacteria including *Haemophilus ducreyi, Shigella sonnei, Escherichia coli* 0157:H7, *Bordetella bronchiseptica* and the Pseudomonas-related species *Burkholderia cenocepacia* [28]. Prophages have been identified in LES [29] and c3719 genomes [30], however BLAST searches of genes from the AES-1R prophage failed to identify homologs in these epidemic strains. There was no discernable pattern of differential expression by genes in this putative accessory region in the AES-1R/1M array comparison conducted in this study. However the upregulation of two non-PAO1 genes belonging to PAGI-5 is interesting as it links AES-1R to the pathogenic features of this gene island found in PA7 and PACS2. PAGI-5-containing strains have been shown to be more virulent than non-PAGI-5 strains in mammalian models [31].

A major objective of this study was to utilize the AES-1R sequence and the PANarray to elucidate the differences and similarities in gene expression between the acute and chronic isolates of AES-1. The finding that certain virulence-related genes were upregulated in chronic infection isolate AES-1M has relevance in areas including biofilm development, transmissibility, antibiotic resistance, pyomelanin-related persistence and lung surfactant secretion (Table 2). The upregulation of *adhA* is required for *P. aeruginosa* biofilm development [32] and biofilms are important in virulence and persistence. We previously reported that AES-1 strains produce significantly larger biofilms than non-epidemic strains [33] thus the upregulated expression of biofilm-related genes in AES-1M would enhance its ability to persist. T3SS genes are generally switched off or downregulated at chronic infection and several including *pcrV*, *pscC* and *pscI* were significantly downregulated in AES-1M; however *pscD*, a regulatory T3SS gene [34] was significantly upregulated in AES-1M. As a functioning regulator, *pscD* may trigger the T3SS and enable AES-1M to infect other CF patients from the environment or transmit to other patients via aerosols from an infected patient. *ppiA* is one of four periplasmic isomerases in *E. coli,* inactivation of which leads to a decreased growth rate and increased susceptibility to certain antibiotics [35]. It has been shown in *E. coli* that *hemE* is the first enzyme transcribed in heme synthesis, and heme synthesis gene expression has been linked to regulation of alginate secretion [36]. While AES-1M appeared non-mucoid on HBA and MHA (see Results), there was significant upregulation of most alginate genes in ASMDM, suggesting that this mucus-like medium triggers their enhanced expression. Alginate is considered a chronic infection virulence factor [12] and is involved in biofilm production and resistance to leukocyte killing [37]. The exception was a significant downregulation of *algC* in AES-1M. *algC* is transcribed separately from the other alginate genes, which are in a single operon under the control of *algD* [38] and has another function as a requirement for lipopolysaccharide (LPS) biosynthesis [39]. Thus downregulation of *algC* would suggest defects in production of A band LPS in these isolates.

Dihydroorotase (*pyrC*-upregulated 12.1fold) catalyzes the third of six enzymatic steps in the biosynthesis of uracil monophosphate (UMP) from glutamine and aspartate precursors while uridylate kinase *pyrH* (upregulated 6.4fold) catalyses the conversion of UMP to UDP. Inhibition of the uracil biosynthetic pathway has been demonstrated to repress biofilms and all three QS pathways (Rhl, Las and Pqs) in *P. aeruginosa* [40] while *Staphylococcus aureus pyrC* mutants showed a fivefold reduction in virulence in a BALB/c mouse competitive systemic infection model and threefold in a non-competitive systemic infection model [41]. Of the genes with probable virulence roles upregulated in AES-1M, *pel* genes are involved in *P. aeruginosa* biofilm formation [42] and enhance a strain's capacity to persist. Expression of *pel* genes by *P. aeruginosa* has also been shown to competitively disrupt *S. aureus* biofilms [43]. The phospholipase C precursor *plcN* is important in extracellular virulence as it degrades phosphatidylcholine, a constituent of lung surfactant [44]. Cardiolipin synthase (*cls*) plays an important role in membrane fluidity and *P. putida cls* mutants have shown increased sensitivity to antibiotics [45].

Other genes upregulated in AES-1M included the putative chemotactic transducer *pctA* (AES_5777 8.2fold) and a *P. aeruginosa* pathogenicity island-1 (PAPI-1) gene (PA14-59980 5.6fold) also found in UCBPP-PA14. PctA is essential for taxis towards sugars, organic acids and L-amino-acids and in the acquisition of nitrogen [46]. The presence of PAPI-1 pathogenicity island genes in AES1 and their upregulation is of great interest due to the role of this island in UCBPP-PA14 virulence [47] and its mode of spread to CF strains [48].

Amongst genes downregulated in AES-1M, *hmgA* and *thxA* stand out. Studies have shown loss of *hmgA* expression induces pyomelanin production, which in turn leads to increased persistence [49] and we have detected downregulation of HmgA at the protein level in AES-1M. Thioredoxin has been shown to be induced in *P. aeruginosa* PAO1 biofilms under strict anaerobic conditions [50], yet in our studies *P. aeruginosa* grown in ASMDM was unlikely to have experienced strictly anaerobic conditions as both surface and subsurface growth has previously been shown to have access to oxygen [24]. Thus AES-1R may be better able to cope with hydrogen peroxide-mediated oxidative stress by induction of the thioredoxin pathway, while chronic AES-1M has adapted by not inducing the host cell-mediated response. Iron uptake is critical in the iron-starved ASMDM and CF sputum [24], thus the synthesis of the siderophores such as pyoverdine and pyochelin would be expected to be high in both acute and chronic isolates. Interestingly, in the pyoverdine biosynthesis operon *pvcABCD, pvcB* was significantly upregulated (3.3fold) while *pvcC* was significantly downregulated (−7.5fold). A recent study suggests *pvcABCD* is required not for pyoverdine production but for dihydroxycumarin expression [51]. *pvcAB* makes an intermediate compound which is oxidized to the catechol form by *pvcCD*. It is possible that the final product is not required by AES-1M, leading to either mutation or downregulation of *pvcC*.

Hoboth et al. [23] compared an early mutator with an end-stage non-mutator phenotype from the same patient, seeing a downregulation of virulence-related genes including T3SS, chemotaxis, QS and flagellin genes. While that study did not use isogenic strains, there are some findings in common with our study. Several chemotaxis transducers (*wspD*, AES_2270 and AES_4899), and T3SS genes (*pcrV*, *pscC* and *pscI*) were downregulated in AES-1M, but most genes in these groups were not differentially expressed. Mutation in any of the chemotaxis genes (*wspABCDEF*) leads to loss of motility and auto-aggregation in *P. aeruginosa* [52]. Therefore the downregulation of *wspD* suggests decreased motility and swarming, possibly leading to less expansion and spread of biofilm microcolonies compared to AES-1R. Our phenotypic data (not shown) demonstrate that AES-1M does swarm less than AES-1R, though not significantly (Pearson's χ^2^ test: p = 0.062) however it swims and twitches significantly more (p = 0.29 and p = 0.015, respectively). With respect to expression of metabolic genes, only one TCA cycle metabolism gene (isocitrate dehydrogenase-*idh*) downregulated in the end-stage mutator [23], was also downregulated in AES-1M, with the remainder not differentially expressed at p<0.05. Possible reasons for the different metabolic profiles include growth media and conditions, and AES-1-strain specific characteristics. Our study used ASMDM, which resembles the composition of lung sputum, and selected the entire biofilm (anaerobic and aerobic growth) for analysis, compared to microaerobic growth in Luria-Bertani broth used in the Hoboth study.

### Conclusions

The sequencing of the AES-1 isolate AES-1R and the use of a broad-capture array has for the first time enabled detection of the expression of AES-1 genes not found in the reference strain PAO1. Furthermore, the use of ASMDM has provided a profile of differential expression of genes under sputum-like conditions. The upregulation of certain virulence-related genes, including biofilm-enhancing, competitive inhibition and persistence genes seen in chronic AES-1M grown in ASMDM offers some explanation for the spread and serious health outcomes associated clinically with AES-1, including person-to-person transmission, more exacerbations and more hospitalisations. In vivo analysis of knockout mutants of differentially expressed genes in persister strains will aid in identifying the factors leading to persistence.

## Materials and Methods

### Strains used in this study

Ethics approval for this study was given by the University of Sydney Human Research Ethics Committee (Protocol Number: X07-0029, Reference Number 6999). The Australian Epidemic Strain-1 (AES-1, previously known as m16, C3789 or PI), is one of two dominant eastern Australian mainland clonal complexes. AES-1R was isolated from a child aged 14 months at the time as the deaths of five CF-infants infected with AES-1 [53]. AES-1M was isolated from the same patient at 11 years 9 months. AES-1 was not eradicated in the patient in the intervening period (D. Armstrong pers. comm.). Written informed consent was obtained by the Royal Children's Hospital Melbourne, and The Southern Health Service, Melbourne.

### Genotyping of AES-1R and AES-1M

AES-1R and AES-1M were genotyped using pulse field gel electrophoresis (PFGE) [54]. Briefly, bacterial cells embedded in agarose plugs were lysed using EC lysis buffer (Sigma-Aldrich Australia), and digested using restriction enzyme *SpeI* to generate a small number (ca. 15–40) of large DNA fragments. After electrophoresis of plugs containing digested DNA on 1.2% w/v agar, band pattern analyses were performed using cluster analysis software (GelComparII™, Applied Maths, Belgium) and the criteria developed by Tenover et al [55] (different genotype if more than a two band difference).

### AES-1R genome sequencing and construction of the PANarray

The AES-1R genome was sequenced using a 454 Genome Sequencer GS20 (Roche Diagnostics, Basel, Switzerland). This produced 598131 reads totalling 58 Mbp, providing approximately nine times coverage of the genome. *De novo* assembly using the Newbler assembler with default parameters, produced 1968 contigs totalling 6.28 Mbp, which were ordered and oriented to the *P. aeruginosa* PAO1 genome [56]. Putative genes were predicted using GeneMarkS 4.6b [57]. The whole genome shotgun sequence of the AES-1R genome (ID: 64619) is available at http://www.ncbi.nlm.nih.gov/nuccore/AFNF00000000. To examine gene homology amongst the eight *P. aeruginosa* genomes (AES-1R, PAO1, PA7/PSPA7, PA14, PACS2, Pa_2192, Pae/PLES and c3719), all gene protein sequences were clustered into orthologous groups using OrthoMCL version 1.4 [58]. Genes were assigned an origin based on the highest identity by BLAST analysis as described on the web based annotation system for prokaryotes of the Victorian Bioinformatics Consortium at Monash University (WASABI) http://vbc.med.monash.edu.au/wasabi/. The AES-1R genes, plus all those from the other seven *P. aeruginosa* genomes were collated for input into the CombiMatrix*®* array design software (CombiMatrix Corp. WA. USA). The resultant non-redundant array contained 12,543 gene probe spots; comprising 12,147 *P. aeruginosa* gene probes (including 1,996 gene probes spotted twice), 326 quality-control probes and 70 probes for non-*P. aeruginosa* genes, including phage and plant genes. Lists of genes unique to each PANarray genome (Table 1) were annotated based on BLAST analysis results, with genes showing an E-value of less than 10^−4^ designated as unique.

### Media preparation and growth conditions

ASMDM was prepared as described previously [24] and triphenyl tetrazolium chloride to a final concentration of 0.025% w/v was added to distinguish respiring cells. Ten milliliters of ASMDM in 20 ml glass screw-cap bottles was inoculated with 50 µl of the diluted culture just under the surface of the media. The cultures were incubated with a loose lid to permit gas exchange at 37°C, and checked for growth every 24 h. ASMDM-grown cells were harvested at 72 h. An uninoculated control was included to detect media contamination.

### Cell preparation and RNA extraction from ASMDM-grown P. aeruginosa

Bacteria and associated biofilm material were removed from the thick surface growth and the deep anaerobic projections at 72h (Fig. 3) for RNA extraction. Cells were washed to increase cell yield and remove debris. Briefly, the bacterial culture (ca. 10 ml) was transferred to a 50 ml Falcon tube and washed 5 times in an equal volume of ice-cold 1×PBS or until the pellet was cleared of non-cellular debris; by pelleting (5 min/5000 *g*/4°C), and re-suspension in fresh ice-cold 1×PBS. All steps were carried out on ice or at 4°C to avoid cell and RNA degradation. Cells were extracted for RNA as previously described [33].

### cDNA synthesis and hybridization

cDNA was synthesized as previously described [59]. cDNA was fragmented using DNaseI and quality-checked using a Bioanalyser (Agilent, Germany). Chosen samples were KreaTech labelled (Agilent) and then hybridized to the Combimatrix® PANarray at the Australian Genome Research Facility (AGRF Ltd, Melbourne) using Cy5 dye. Hybridization conditions were as previously described [33], [59].

### Replicates for microarray analysis

AES-1R, AES-1M were arrayed as biological duplicates (same isolate, different culture, different RNA extraction, and different microarray) giving a total of four data sets, to assess biological variability at the level of culture. Substitution of different biological (culture) replicates was conducted for AES-1R to test for variability, and these had little or no effect on the prediction of differentially expressed genes.

### Data analysis

The raw data were re-scaled to account for differences in individual hybridization intensities. Background corrected data were imported into Partek© software, version 6.4 2010 (Partek Inc.), and quantile normalisation (Bolstad et al, 2003) was carried out on the four AES-1 data sets. Data for each pair of biological duplicates were averaged and the average of the acute and chronic isolates was used to determine differential-expression. Genes that were differentially expressed were determined by an ANOVA model with a cut-off value of p = 0.05. All microarray data is MIAME compliant and both the raw and normalized data have been deposited in the MIAME compliant database Gene Expression Omnibus (GEO) http://www.ncbi.nlm.nih.gov/projects/geo under platform accession number GPL13324/Series GSE28152.

### Gene expression by quantitative PCR

Thirteen genes mainly virulence-related genes that showed significant differential expression by microarray (*adhA*, *algC*, AES_6147, AES_2005, *pyrC, pelB*, *cls*, *algD*. *ppiA*, *pscD*, *hemE*, *pvcC* and *hmgA*) were also checked for expression by quantitative SYBR-green-PCR (qPCR) using a Rotor-Gene6000 Real-Time amplification system (Qiagen), and performed on cDNA synthesised from the microarray RNA or synthesised from RNA extracted from later equivalent experiments. Genes were chosen based on high differential expression and/or association with virulence (Tables 2 and 3). Oligonucleotide primers were designed using Primer Express (Applied Biosystems). cDNA was synthesised as described (Invitrogen), by reverse transcription (RT) using 50U SuperScriptII RT (Invitrogen) and 1 µg total RNA.

## Supporting Information

Table S1 Genes unique* to ***P. aeruginosa*** **AES-1R based on BLAST analysis of the AES-1R genome.**
(DOC)

Table S2 **Genes differentially expressed between** ***P. aeruginosa*** **AES-1R and AES-1M grown in ASMDM (p<0.05).**
(DOC)

Table S3 Genes without homologues in PAO1* that were differentially expressed between ***P. aeruginosa*** **AES-1R and AES-1M grown in ASMDM (p<0.05).**
(DOC)
