# Supplementary material for: Pseudomonas aeruginosa AES-1 Exhibits Increased Virulence Gene Expression during Chronic Infection of Cystic Fibrosis Lung
Source: PLoS One. 2011 Sep 15;6(9):e24526. doi: 10.1371/journal.pone.0024526 (PMC3174184; doi:10.1371/journal.pone.0024526)
Supplement: Table S1 — Genes unique* to P. aeruginosa AES-1R based on BLAST analysis of the AES-1R genome. (DOC) [file pone.0024526.s001.doc]

**Supplementary Table 1: Genes unique* to *P. aeruginosa* AES-1 based on BLAST analysis of the AES-1 genome.**

| **Gene ID** | **Length (amino acids)** | **Description** | **Location** |
| --- | --- | --- | --- |
| AES_0007 | 117 | hypothetical protein |  |
| AES_0067 | 589 | hypothetical protein | OM |
| AES_0068 | 43 | hypothetical protein | CYT |
| AES_0070 | 45 | hypothetical protein |  |
| AES_0072 | 59 | hypothetical protein |  |
| AES_0177 | 43 | hypothetical protein |  |
| AES_0215 | 39 | hypothetical protein |  |
| AES_0217 | 181 | hypothetical protein |  |
| AES_0225 | 39 | hypothetical protein |  |
| AES_0266 | 76 | hypothetical protein |  |
| AES_0364 | 76 | hypothetical protein |  |
| AES_0382 | 44 | putative esterase | UNK |
| AES_0446 | 57 | hypothetical protein |  |
| AES_0449 | 46 | hypothetical protein |  |
| AES_0500 | 41 | hypothetical protein | CYT |
| AES_0603 | 39 | hypothetical protein |  |
| AES_0660 | 39 | putative glutamate synthase, large subunit | IM |
| AES_0699 | 46 | hypothetical protein |  |
| AES_0752 | 71 | hypothetical protein |  |
| AES_0771 | 41 | hypothetical protein |  |
| AES_0803 | 195 | hypothetical protein | CYT |
| AES_0826 | 92 | hypothetical protein |  |
| AES_0833 | 135 | TrbG-like protein | IM |
| AES_0897 | 73 | hypothetical protein |  |
| AES_0901 | 42 | hypothetical protein |  |
| AES_0918 | 61 | hypothetical protein |  |
| AES_0929 | 48 | hypothetical protein |  |
| AES_1039 | 49 | hypothetical protein |  |
| AES_1066 | 52 | hypothetical protein |  |
| AES_1220 | 102 | hypothetical protein |  |
| AES_1226 | 99 | anthranilate synthase component II *trpG* | CYT |
| AES_1254 | 38 | hypothetical protein |  |
| AES_1268 | 42 | hypothetical protein |  |
| AES_1290 | 47 | hypothetical protein |  |
| AES_1342 | 461 | hypothetical protein |  |
| AES_1353 | 46 | hypothetical protein |  |
| AES_1354 | 474 | hypothetical protein |  |
| AES_1378 | 43 | hypothetical protein |  |
| AES_1410 | 70 | hypothetical protein |  |
| AES_1412 | 42 | hypothetical protein |  |
| AES_1431 | 50 | hypothetical protein | CYT |
| AES_1455 | 38 | hypothetical protein |  |
| AES_1464 | 101 | hypothetical protein |  |
| AES_1518 | 43 | hypothetical protein |  |
| AES_1523 | 71 | hypothetical protein |  |
| AES_1524 | 731 | hypothetical protein | CYT |
| AES_1554 | 404 | DNA cytosine methyltransferase |  |
| AES_1584 | 40 | hypothetical protein |  |
| AES_1625 | 48 | hypothetical protein |  |
| AES_1729 | 55 | hypothetical protein |  |
| AES_1754 | 52 | hypothetical protein |  |
| AES_1797 | 51 | hypothetical protein |  |
| AES_1836 | 45 | hypothetical protein |  |
| AES_1856 | 87 | hypothetical protein |  |
| AES_1860 | 43 | hypothetical protein |  |
| AES_1887 | 49 | hypothetical protein |  |
| AES_1909 | 42 | hypothetical protein |  |
| AES_1919 | 41 | hypothetical protein |  |
| AES_1959 | 41 | hypothetical protein | CYT |
| AES_1973 | 148 | hypothetical protein |  |
| AES_1979 | 46 | hypothetical protein |  |
| AES_2001 | 46 | hypothetical protein |  |
| AES_2090 | 47 | hypothetical protein |  |
| AES_2101 | 91 | hypothetical protein |  |
| AES_2127 | 60 | hypothetical protein |  |
| AES_2152 | 61 | hypothetical protein |  |
| AES_2191 | 146 | putative AMP-binding enzyme | CYT |
| AES_2208 | 40 | hypothetical protein |  |
| AES_2225 | 49 | hypothetical protein |  |
| AES_2227 | 52 | hypothetical protein |  |
| AES_2244 | 234 | Hypothetical protein | UNK |
| AES_2250 | 38 | hypothetical protein |  |
| AES_2376 | 38 | hypothetical protein |  |
| AES_2429 | 262 | Heme exporter protein CcmA *ccmA* | IM |
| AES_2430 | 38 | hypothetical protein | CYT |
| AES_2439 | 127 | Uncharacterized conserved protein |  |
| AES_2440 | 118 | Mu-like prophage protein gp16 |  |
| AES_2446 | 79 | hypothetical protein |  |
| AES_2451 | 56 | hypothetical protein |  |
| AES_2455 | 190 | hypothetical protein |  |
| AES_2520 | 39 | hypothetical protein |  |
| AES_2530 | 88 | hypothetical protein | CYT |
| AES_2531 | 38 | hypothetical protein |  |
| AES_2574 | 50 | putative transporter | IM |
| AES_2578 | 56 | hypothetical protein |  |
| AES_2627 | 78 | hypothetical protein |  |
| AES_2696 | 56 | hypothetical protein |  |
| AES_2709 | 41 | hypothetical protein |  |
| AES_2763 | 38 | hypothetical protein |  |
| AES_2787 | 45 | hypothetical protein |  |
| AES_2835 | 46 | hypothetical protein |  |
| AES_2947 | 39 | hypothetical protein |  |
| AES_2951 | 55 | hypothetical protein |  |
| AES_2985 | 38 | hypothetical protein | CYT |
| AES_3049 | 38 | hypothetical protein |  |
| AES_3092 | 40 | hypothetical protein |  |
| AES_3173 | 40 | hypothetical protein |  |
| AES_3178 | 41 | hypothetical protein |  |
| AES_3216 | 45 | hypothetical protein |  |
| AES_3220 | 43 | hypothetical protein |  |
| AES_3278 | 43 | hypothetical protein |  |
| AES_3312 | 49 | hypothetical protein |  |
| AES_3322 | 48 | hypothetical protein |  |
| AES_3323 | 43 | hypothetical protein |  |
| AES_3449 | 38 | putative 4-alpha-glucanotransferase | CYT |
| AES_3465 | 63 | hypothetical protein |  |
| AES_3466 | 42 | hypothetical protein |  |
| AES_3499 | 45 | putative NADP-dependent oxidoreductase† | CYT |
| AES_3500 | 294 | putative NADP-dependent oxidoreductase† | CYT |
| AES_3609 | 41 | hypothetical protein |  |
| AES_3689 | 44 | hypothetical protein |  |
| AES_3737 | 60 | hypothetical protein |  |
| AES_3781 | 43 | hypothetical protein |  |
| AES_3798 | 42 | hypothetical protein |  |
| AES_3801 | 42 | hypothetical protein |  |
| AES_3817 | 51 | hypothetical protein |  |
| AES_3868 | 86 | hypothetical protein |  |
| AES_3880 | 57 | hypothetical protein | CYT |
| AES_4078 | 39 | hypothetical protein |  |
| AES_4115 | 44 | hypothetical protein |  |
| AES_4128 | 77 | hypothetical protein |  |
| AES_4132 | 39 | hypothetical protein |  |
| AES_4145 | 44 | hypothetical protein |  |
| AES_4167 | 41 | hypothetical protein |  |
| AES_4179 | 38 | hypothetical protein |  |
| AES_4197 | 43 | hypothetical protein |  |
| AES_4225 | 56 | hypothetical protein |  |
| AES_4264 | 50 | hypothetical protein | CYT |
| AES_4269 | 44 | hypothetical protein | CYT |
| AES_4375 | 586 | hypothetical protein |  |
| AES_4382 | 39 | hypothetical protein |  |
| AES_4388 | 163 | hypothetical protein |  |
| AES_4409 | 42 | hypothetical protein |  |
| AES_4497 | 129 | putative integrase | UNK |
| AES_4502 | 41 | hypothetical protein |  |
| AES_4503 | 158 | hypothetical protein |  |
| AES_4504 | 70 | hypothetical protein | CYT |
| AES_4505 | 226 | hypothetical protein |  |
| AES_4506 | 115 | hypothetical protein | CYT |
| AES_4551 | 42 | hypothetical protein |  |
| AES_4563 | 39 | hypothetical protein |  |
| AES_4564 | 49 | hypothetical protein |  |
| AES_4589 | 47 | hypothetical protein |  |
| AES_4599 | 44 | hypothetical protein |  |
| AES_4648 | 40 | hypothetical protein |  |
| AES_4721 | 44 | hypothetical protein |  |
| AES_4744 | 38 | hypothetical protein |  |
| AES_4772 | 50 | hypothetical protein |  |
| AES_4776 | 51 | hypothetical protein |  |
| AES_4777 | 104 | hypothetical protein | CYT |
| AES_4778 | 233 | DNA polymerase III, beta chain *dnaN* | CYT |
| AES_4781 | 72 | hypothetical protein |  |
| AES_4827 | 98 | hypothetical protein |  |
| AES_4840 | 40 | hypothetical protein | CYT |
| AES_4905 | 42 | hypothetical protein | CYT |
| AES_5038 | 113 | putative ornithine cyclodeaminase | CYT |
| AES_5061 | 71 | hypothetical protein |  |
| AES_5119 | 40 | hypothetical protein |  |
| AES_5165 | 42 | hypothetical protein |  |
| AES_5245 | 78 | hypothetical protein |  |
| AES_5260 | 43 | hypothetical protein |  |
| AES_5288 | 42 | hypothetical protein |  |
| AES_5300 | 39 | hypothetical protein |  |
| AES_5306 | 179 | 2-isopropylmalate synthase *leuA* | UNK |
| AES_5318 | 43 | hypothetical protein |  |
| AES_5333 | 45 | hypothetical protein |  |
| AES_5355 | 41 | hypothetical protein |  |
| AES_5399 | 69 | hypothetical protein |  |
| AES_5438 | 59 | hypothetical protein |  |
| AES_5472 | 65 | hypothetical protein |  |
| AES_5522 | 40 | hypothetical protein |  |
| AES_5527 | 39 | hypothetical protein |  |
| AES_5532 | 154 | polyhydroxyalkanoate synthesis protein PhaF *phaF*† | IM |
| AES_5535 | 49 | hypothetical protein |  |
| AES_5573 | 42 | hypothetical protein |  |
| AES_5576 | 63 | hypothetical protein |  |
| AES_5578 | 107 | hypothetical protein |  |
| AES_5620 | 50 | hypothetical protein |  |
| AES_5677 | 38 | hypothetical protein |  |
| AES_5694 | 71 | pyochelin synthetase PchF *pchF* | CYT |
| AES_5707 | 95 | hypothetical protein |  |
| AES_5777 | 62 | hypothetical protein |  |
| AES_5780 | 52 | hypothetical protein |  |
| AES_5840 | 63 | hypothetical protein |  |
| AES_5846 | 45 | hypothetical protein |  |
| AES_6025 | 48 | hypothetical protein |  |
| AES_6027 | 67 | hypothetical protein |  |
| AES_6033 | 53 | hypothetical protein |  |
| AES_6040 | 185 | putative ABC transporter, ATP-binding protein | IM |
| AES_6246 | 45 | hypothetical protein |  |
| AES_6306 | 52 | hypothetical protein |  |
| AES_6330 | 46 | hypothetical protein |  |
| AES_6392 | 70 | hypothetical protein | CYT |
| AES_6417 | 50 | hypothetical protein |  |
| AES_6426 | 50 | hypothetical protein |  |
| AES_6507 | 50 | hypothetical protein |  |
| AES_6518 | 39 | hypothetical protein |  |
| AES_6594 | 45 | hypothetical protein |  |
| AES_6671 | 73 | hypothetical protein |  |
| AES_6731 | 39 | hypothetical protein |  |
| AES_6802 | 48 | hypothetical protein |  |
| AES_6820 | 40 | hypothetical protein |  |
| AES_6835 | 70 | hypothetical protein |  |
| AES_6851 | 41 | hypothetical protein |  |
| AES_6895 | 151 | putative hydrolase | CYT |
| AES_6952 | 50 | hypothetical protein |  |
| AES_6966 | 56 | hypothetical protein |  |
| AES_6968 | 195 | hypothetical protein | CYT |
| AES_6969 | 222 | hypothetical protein | UNK |
| AES_6970 | 466 | putative DNA helicase | CYT |
| AES_6972 | 252 | Putative phage baseplate assembly protein | CYT |
| AES_6973 | 127 | Bacteriophage P2-related tail formation protein *gpI* | CYT |
| AES_6974 | 46 | hypothetic**a**l protein |  |
| AES_6976 | 189 | hypothetical protein | CYT |
| AES_6978 | 300 | Recombinational DNA repair protein *recT* |  |
| AES_6979 | 241 | Phage-related protein, predicted endonuclease | CYT |
| AES_6982 | 156 | hypothetical protein |  |
| AES_6983 | 74 | hypothetical protein |  |
| AES_6984 | 48 | hypothetical protein |  |
| AES_6986 | 380 | hypothetical protein |  |
| AES_6987 | 634 | hypothetical protein | OM |
| AES_6988 | 74 | hypothetical protein |  |
| AES_6989 | 349 | Site-specific DNA methylase *dcm* | CYT |
| AES_6990 | 1214 | hypothetical protein | OM |
| AES_6991 | 65 | hypothetical protein |  |
| AES_6992 | 281 | Putative integrase | UNK |
| AES_6996 | 96 | conserved hypothetical protein | CYT |
| AES_6997 | 326 | hypothetical protein | CYT |
| AES_7000 | 40 | hypothetical protein |  |
| AES_7001 | 403 | hypothetical protein |  |
| AES_7002 | 1141 | putative exonuclease *sbcC* | OM |
| AES_7003 | 105 | hypothetical protein |  |
| AES_7004 | 324 | hypothetical protein | CYT |
| AES_7005 | 85 | hypothetical protein |  |
| AES_7006 | 391 | Phage-related protein, predicted endonuclease | CYT |
| AES_7007 | 40 | hypothetical protein |  |
| AES_7008 | 48 | hypothetical protein |  |
| AES_7010 | 499 | Mu-like prophage protein gp29 |  |
| AES_7011 | 467 | Mu-like prophage FluMu protein gp28 |  |
| AES_7012 | 38 | hypothetical protein |  |
| AES_7017 | 44 | hypothetical protein |  |
| AES_7020 | 42 | hypothetical protein |  |
| AES_7021 | 53 | hypothetical protein |  |
| AES_7022 | 62 | hypothetical protein |  |
| AES_7025 | 49 | hypothetical protein | CYT |
| AES_7026 | 76 | putative zinc finger protein | CYT |
| AES_7027 | 93 | hypothetical protein |  |
| AES_7028 | 52 | hypothetical protein |  |
| AES_7029 | 56 | hypothetical protein | CYT |
| AES_7030 | 65 | hypothetical protein | CYT |
| AES_7031 | 216 | SOS-response transcriptional repressors (RecA-mediated autopeptidases) *lexA* | CYT |
| AES_7032 | 93 | hypothetical protein | CYT |
| AES_7033 | 155 | hypothetical protein | CYT |
| AES_7034 | 44 | hypothetical protein |  |
| AES_7035 | 44 | hypothetical protein |  |
| AES_7036 | 172 | hypothetical protein |  |
| AES_7041 | 52 | hypothetical protein |  |
| AES_7044 | 70 | hypothetical protein | CYT |
| AES_7045 | 54 | hypothetical protein |  |
| AES_7047 | 46 | hypothetical protein |  |
| AES_7048 | 46 | hypothetical protein |  |
| AES_7049 | 98 | putative baseplate assembly protein W | UNK |
| AES_7050 | 136 | hypothetical protein |  |
| AES_7051 | 39 | hypothetical protein |  |
| AES_7052 | 283 | Type II secretory pathway, component ExeA | CYT |
| AES_7053 | 39 | putative baseplate assembly protein V | UNK |
| AES_7055 | 132 | DNA modification methylase |  |
| AES_7056 | 95 | hypothetical protein |  |
| AES_7057 | 696 | hypothetical protein | CYT |
| AES_7058 | 515 | hypothetical protein |  |
| AES_7059 | 81 | hypothetical protein |  |
| AES_7060 | 62 | hypothetical protein | CYT |
| AES_7061 | 266 | hypothetical protein |  |
| AES_7062 | 48 | hypothetical protein |  |
| AES_7063 | 209 | hypothetical protein |  |
| AES_7064 | 91 | hypothetical protein |  |
| AES_7065 | 100 | hypothetical protein |  |
| AES_7066 | 160 | hypothetical protein | CYT |
| AES_7067 | 46 | hypothetical protein | CYT |
| AES_7068 | 67 | hypothetical protein |  |
| AES_7069 | 82 | hypothetical protein | CYT |
| AES_7071 | 212 | DNA replication protein *dnaC* | CYT |
| AES_7073 | 38 | hypothetical protein |  |
| AES_7075 | 43 | hypothetical protein |  |
| AES_7076 | 896 | putative tail length determinator protein | OM |
| AES_7077 | 290 | Hypothetical protein |  |
| AES_7078 | 239 | Putative phage late control gene D protein | UNK |
| AES_7079 | 76 | hypothetical protein | CYT |
| AES_7080 | 162 | putative phage tail tube protein | UNK |
| AES_7081 | 476 | putative phage tail sheath protein | UNK |
| AES_7082 | 79 | hypothetical protein |  |
| AES_7083 | 160 | hypothetical protein |  |
| AES_7084 | 144 | Mu-like prophage protein gp36 | CYT |
| AES_7085 | 111 | hypothetical protein |  |
| AES_7086 | 112 | hypothetical protein |  |
| AES_7087 | 308 | hypothetical protein | CYT |
| AES_7088 | 103 | hypothetical protein | CYT |
| AES_7089 | 293 | hypothetical protein |  |
| AES_7090 | 161 | putative phosphotransferase system, fructose-specific component |  |
| AES_7101 | 43 | hypothetical protein |  |
| AES_7102 | 274 | Protease subunit of ATP-dependent Clp proteases *clpP* |  |
| AES_7103 | 121 | hypothetical protein |  |
| AES_7105 | 75 | hypothetical protein |  |
| AES_7106 | 56 | hypothetical protein |  |
| AES_7111 | 845 | hypothetical protein |  |
| AES_7112 | 197 | hypothetical protein |  |
| AES_7113 | 383 | hypothetical protein |  |
| AES_7114 | 780 | hypothetical protein |  |
| AES_7116 | 92 | hypothetical protein | CYT |
| AES_7117 | 202 | hypothetical protein |  |
| AES_7119 | 72 | hypothetical protein |  |
| AES_7120 | 715 | putative tail fiber protein | ExM |
| AES_7122 | 347 | hypothetical protein | CYT |
| AES_7123 | 368 | hypothetical protein | CYT |
| AES_7124 | 132 | hypothetical protein |  |
| AES_7125 | 137 | hypothetical protein |  |
| AES_7126 | 220 | hypothetical protein | CYT |
| AES_7128 | 75 | hypothetical protein |  |
| AES_7130 | 51 | hypothetical protein |  |
| AES_7131 | 172 | hypothetical protein | CYT |
| AES_7132 | 47 | hypothetical protein | CYT |
| AES_7133 | 216 | hypothetical protein |  |
| AES_7134 | 202 | hypothetical protein |  |
| AES_7136 | 278 | Hypothetical protein |  |
| AES_7137 | 38 | hypothetical protein |  |
| AES_7141 | 67 | hypothetical protein |  |
| AES_7147 | 39 | hypothetical protein |  |
| AES_7148 | 157 | hypothetical protein |  |
| AES_7149 | 86 | hypothetical protein |  |
| AES_7150 | 84 | hypothetical protein |  |
| AES_7151 | 171 | Phage-related lysozyme (muraminidase) |  |
| AES_7152 | 56 | hypothetical protein |  |
| AES_7165 | 217 | hypothetical protein | CYT |
| AES_7166 | 92 | hypothetical protein | CYT |
| AES_7167 | 113 | conserved hypothetical protein | CYT |
| AES_7170 | 50 | hypothetical protein |  |
| AES_7175 | 11 | translated portion of tmRNA gene *ssrA* |  |
| AES_7176 | 184 | hypothetical protein |  |
| AES_7185 | 36 | hypothetical protein | CYT |

* Genes having no homologs with an E-value greater than 10^-4^ were designated as unique**.**

† Genes with hypothetical protein homologs in other *P. aeruginosa* genomes
