# Supplementary material for: Pseudomonas aeruginosa AES-1 Exhibits Increased Virulence Gene Expression during Chronic Infection of Cystic Fibrosis Lung
Source: PLoS One. 2011 Sep 15;6(9):e24526. doi: 10.1371/journal.pone.0024526 (PMC3174184; doi:10.1371/journal.pone.0024526)
Supplement: Table S2 — Genes differentially expressed between P. aeruginosa AES-1R and AES-1M grown in ASMDM (p<0.05). (DOC) [file pone.0024526.s002.doc]

**Supplementary Table 2: Genes differentially expressed between *P. aeruginosa* AES-1R and AES-1M (p<0.05).**

| **Gene ID Description** | **Fold Change§** |
| --- | --- |
| AES_0046 *betC* choline sulfatase | 3.0 |
| AES_0049 putative dioxygenase | -1.6 |
| AES_0066 conserved hypothetical protein | -2.5 |
| AES_0071 hypothetical protein | -4.2 |
| AES_0073 *amiE* aliphatic amidase | -2.2 |
| AES_0093 conserved hypothetical protein | -2.2 |
| AES_0104 conserved hypothetical protein | 3.5 |
| AES_0127 putative lipoprotein | -1.9 |
| AES_0230 putative short-chain dehydrogenase | -2.2 |
| AES_0232 putative transmembrane sensor | -3.1 |
| AES_0249 putative RND efflux membrane fusion protein precursor | 3.4 |
| AES_0254 putative efflux transmembrane protein | 1.4 |
| AES_0266 hypothetical protein | 11.1 |
| AES_0276 putative phosphoesterase | 6.1 |
| AES_0277 *folA* dihydrofolate reductase | -3.4 |
| AES_0284 hypothetical protein | -2.6 |
| AES_0324 conserved hypothetical protein | 2.0 |
| AES_0326 putative lipoprotein | 3.4 |
| AES_0332 putative hypoxanthine phosphoribosyltransferase | -2.1 |
| AES_0335 hypothetical protein | -2.3 |
| AES_0342 hypothetical protein | 2.5 |
| AES_0353 *mtlD* mannitol dehydrogenase | -3.1 |
| AES_0365 putative alkylhydroperoxidase | -1.8 |
| AES_0386 Metal-dependent amidase/aminoacylase/carboxypeptidase | 3.9 |
| AES_0405 putative amino acid permease | 3.1 |
| AES_0415 *spuB* glutamine synthetase | 3.5 |
| AES_0418 putative ATP-binding component of ABC transporter | -2.8 |
| AES_0428 *pcaC* gamma-carboxymuconolactone decarboxylase | 1.4 |
| AES_0439 putative ABC transporter | 2.7 |
| AES_0453 *pvcC* pyoverdine biosynthesis protein | -7.5 |
| AES_0454 *pvcB* pyoverdine biosynthesis protein | 3.3 |
| AES_0458 hypothetical protein | 3.3 |
| AES_0462 putative hydrolase | 3.6 |
| AES_0520 conserved hypothetical protein | -1.6 |
| AES_0528 putative transcriptional regulator, GntR family | 2.2 |
| AES_0540 amidase family protein | 12.2 |
| AES_0542 putative decarboxylase | 1.9 |
| AES_0576 Hypothetical protein | -2.1 |
| AES_0618 *potA* polyamine transport protein | -2.1 |
| AES_0634 conserved hypothetical protein | -2.0 |
| AES_0655 putative amino acid/amine transport protein | -5.0 |
| AES_0678 *ilvA1* threonine dehydratase, biosynthetic | 9.7 |
| AES_0679 *rpiA* ribose 5-phosphate isomerase | -2.0 |
| AES_0715 putative oxidoreductase, GMC family | 6.3 |
| AES_0723 putative transcriptional regulator, TetR family | 2.6 |
| AES_0754 *gbt* glycine betaine transmethylase | -2.2 |
| AES_0763 conserved hypothetical protein | 3.5 |
| AES_0778 cystathionine beta-synthase | 1.3 |
| AES_0781 *pilU* twitching motility protein | -3.3 |
| AES_0786 *yqgF* putative ribonuclease | 8.0 |
| AES_0787 putative transcriptional activator of alginate synthesis | 1.3 |
| AES_0797  *chpA* component of chemotactic signal transduction system | 3.2 |
| AES_0808 putative cytochrome b561 | 2.1 |
| AES_0818 *mexC* multidrug efflux RND membrane fusion protein | 4.2 |
| AES_0820 *oprJ* outer membrane protein | -2.1 |
| AES_0822 hypothetical protein | -3.6 |
| AES_0852 putative outer membrane ferric siderophore receptor | -2.3 |
| AES_0861 *amaB* N-carbamoyl-beta-alanine amidohydrolase | -9.0 |
| AES_0868 *gcdH* glutaryl-CoA dehydrogenase | -2.3 |
| AES_0905 putative ATPase | -3.3 |
| AES_0941 conserved hypothetical protein | 9.6 |
| AES_0954 FAD-containing subunit of NADH dehydrogenase | 1.6 |
| AES_0955 putative hydrolase | -2.5 |
| AES_0957 putative hydroxamate-type ferrisiderophore receptor | 1.8 |
| AES_0961 putative glutathione S-transferase | 2.6 |
| AES_0963 putative transcriptional regulator | -1.7 |
| AES_1011 putative acyl-CoA dehydrogenase | -1.5 |
| AES_1020 hypothetical protein | -2.1 |
| AES_1037 putative nuclease | 2.7 |
| AES_1048 *fruK* 1-phosphofructokinase | 4.2 |
| AES_1058 thiosulfate sulfurtransferase | 3.4 |
| AES_1075 *norB* nitric-oxide reductase subunit B | 1.4 |
| AES_1100 hypothetical protein | 4.9 |
| AES_1125 *algF* alginate O-acetyltransferase | 3.6 |
| AES_1132 *algE* Alginate outer membrane protein | 2.6 |
| AES_1135 *alg8* alginate biosynthesis protein | 10.7 |
| AES_1136 *algD* GDP-mannose 6-dehydrogenase | 3.1 |
| AES_1156 putative hydroxypyruvate isomerase | -3.2 |
| AES_1186 conserved hypothetical protein | -2.5 |
| AES_1207 putative two-component response regulator | 2.8 |
| AES_1225 *trpG* anthranilate synthase component II | -2.1 |
| AES_1235 putative phage tail tube protein | 2.4 |
| AES_1276 hypothetical protein | 2.6 |
| AES_1282 hypothetical protein | -1.7 |
| AES_1316 *exbD2* transport protein | 1.6 |
| AES_1317 *exbB2* transport protein | -2.8 |
| AES_1325 probable transcriptional regulator | -2.0 |
| AES_1381 conserved hypothetical protein | -3.0 |
| AES_1393 putative outer membrane porin | 3.6 |
| AES_1400 hypothetical protein | 4.0 |
| AES_1405 hypothetical protein | 1.5 |
| AES_1406 *ada* O6-methylguanine-DNA methyltransferase | -3.6 |
| AES_1408 hypothetical protein | 1.7 |
| AES_1418 hypothetical protein | 5.3 |
| AES_1439 *mucD* serine protease MucD precursor | -2.5 |
| AES_1453 conserved hypothetical protein | 3.4 |
| AES_1474 hypothetical protein | -5.6 |
| AES_1492 putative oxidoreductase | -3.4 |
| AES_1498 hypothetical protein | 4.7 |
| AES_1500 hypothetical protein | -1.8 |
| AES_1502 probable haloacid dehalogenase | 21.6 |
| AES_1517 hypothetical protein | 2.4 |
| AES_1520 hypothetical protein | -1.9 |
| AES_1534 *oruR* transcriptional regulator | 2.2 |
| AES_1546 probable glycosyl transferase | 4.2 |
| AES_1601 hypothetical protein | 2.1 |
| AES_1605 ring-hydroxylating dioxygenase, large terminal subunit | -1.8 |
| AES_1617 hypothetical protein | -7.1 |
| AES_1624 conserved hypothetical protein | -2.2 |
| AES_1644  *rmlA* glucose-1-phosphate thymidylyltransferase | 1.8 |
| AES_1649 probable C4-dicarboxylate transporter | -5.5 |
| AES_1657 conserved hypothetical protein | 12.2 |
| AES_1666  *cysQ* 3'(2'),5'-bisphosphate nucleotidase | 3.1 |
| AES_1667 putative beta-ketoacyl synthase | 3.2 |
| AES_1680 *pgi* glucose-6-phosphate isomerase | -4.7 |
| AES_1706 *alaS* alanyl-tRNA synthetase | -4.6 |
| AES_1723 hypothetical protein | -1.5 |
| AES_1752 hypothetical protein | -2.9 |
| AES_1774 *proS* prolyl-tRNA synthetase | -2.4 |
| AES_1779 probable cold-shock protein | -2.2 |
| AES_1780 probable dna-binding stress protein | 2.5 |
| AES_1797 hypothetical protein | -1.9 |
| AES_1817  *pqsC* quinolone signal biosynthesis protein | -2.2 |
| AES_1821 *cupC3* fimbrial biogenesis usher protein | -4.6 |
| AES_1828 *bcp* bacterioferritin co-migratory protein | 2.2 |
| AES_1837 Putative metal-dependent hydrolase | -2.2 |
| AES_1858 Hypothetical protein | -3.4 |
| AES_1889 Probable transporter | -2.1 |
| AES_1901 Conserved hypothetical protein | -2.6 |
| AES_1915 hypothetical protein | -6.6 |
| AES_1925 Putative branched-chain amino acid ABC transporter, ATP-binding | 6.1 |
| AES_1926 Putative ranched-chain amino acid ABC transporter, ATP-binding | -6.6 |
| AES_1951 Hypothetical protein | -1.6 |
| AES_1984 putative FMNH2-dependent monooxygenase | 5.8 |
| AES_2005 putative sigma-54 dependent transcriptional regulator | 6.5 |
| AES_2025 conserved hypothetical protein | 3.4 |
| AES_2065 hypothetical protein | -2.6 |
| AES_2073 hypothetical protein | 5.9 |
| AES_2079 Putative Fe-S-cluster | 13.2 |
| AES_2110 conserved hypothetical protein | -1.9 |
| AES_2124 hypothetical protein | -3.8 |
| AES_2153 putative dimethylarginine dimethylaminohydrolase | 11.3 |
| AES_2176 probable porin | 2.3 |
| AES_2195 conserved hypothetical protein | -6.2 |
| AES_2222 putative transcriptional regulator | 3.9 |
| AES_2245 conserved hypothetical protein | -2.6 |
| AES_2246 putative outer membrane protein | -1.5 |
| AES_2260 putative ATP-binding component of ABC transporter | 8.4 |
| AES_2271 putative chemotaxis transducer | -8.4 |
| AES_2275 hypothetical protein | 2.1 |
| AES_2315 *cobD* cobalamin biosynthetic protein | -2.8 |
| AES_2321 putative alpha-ribazole-5'-phosphate phosphatase | 1.2 |
| AES_2323 *cobV* cobalamin (5'-phosphate) synthase | -1.4 |
| AES_2333  *sseA* 3-mercaptopyruvate sulfurtransferase | 3.5 |
| AES_2354 putative transcriptional regulator, LysR family | -1.8 |
| AES_2366 *cyoE* cytochrome o ubiquinol oxidase protein | -2.0 |
| AES_2386 *morB* morphinone reductase | 2.4 |
| AES_2391 probable xenobiotic reductase B | 4.5 |
| AES_2393 putative transcriptional regulator | 1.7 |
| AES_2413 putative short-chain dehydrogenase | 5.0 |
| AES_2425 conserved hypothetical protein | 3.5 |
| AES_2451 hypothetical protein | -8.1 |
| AES_2458 putative ABC transporter ATP-binding component | -6.6 |
| AES_2461 hypothetical protein | 20.5 |
| AES_2475 hypothetical protein | -2.5 |
| AES_2493 *lasI* autoinducer synthesis protein | 2.3 |
| AES_2504 putative two-component sensor | 2.3 |
| AES_2509 *fliR* flagellar biosynthetic protein | 2.2 |
| AES_2528 *cheZ* chemotaxis protein | -2.7 |
| AES_2542 putative ribosomal-protein-serine acetyltransferase | -5.9 |
| AES_2601 putative permease | 3.7 |
| AES_2606 putative chromosome segregation protein | -2.4 |
| AES_2609  *lig* DNA ligase | 4.1 |
| AES_2617 conserved hypothetical protein | -4.6 |
| AES_2618 *dnaX* DNA polymerase subunits gamma and tau | 2.9 |
| AES_2630 putative hydrolase | -1.9 |
| AES_2633 conserved hypothetical protein | 16.3 |
| AES_2656 putative transcriptional regulator, LysR family | -1.6 |
| AES_2708  *fabA* beta-hydroxydecanoyl-ACP dehydrase | -1.7 |
| AES_2715 putative acetyltransferase, GNAT family | -5.3 |
| AES_2743 *kdpB* potassium-transporting ATPase, B chain | -3.3 |
| AES_2771 putative transcriptional regulator, MarR family | 2.5 |
| AES_2773 putative aminotransferase | -5.5 |
| AES_2788 putative ClpA/B-type protease | -2.8 |
| AES_2789 putative outer membrane protein | 1.6 |
| AES_2808 *folE1* GTP cyclohydrolase I precursor | 1.5 |
| AES_2843 putative protein in type III secretion | 1.8 |
| AES_2852 *pcrV* type III secretion protein | -2.6 |
| AES_2863 *pscC* Type III secretion outer membrane protein precursor | -3.4 |
| AES_2864 *pscD* type III export protein | 3.2 |
| AES_2867 *pscI* type III export protein | -3.0 |
| AES_2882 putative glutaredoxin | -2.1 |
| AES_2883 conserved hypothetical protein | -2.9 |
| AES_2888 putative permease | -1.9 |
| AES_2895 hypothetical protein | -2.7 |
| AES_2950 *acnB* aconitate hydratase 2 | 1.7 |
| AES_2952 Hypothetical protein | 3.4 |
| AES_2966 Hypothetical protein | -3.0 |
| AES_2983 *ppiA* peptidyl-prolyl cis-trans isomerase A | 2.5 |
| AES_3003 putative Orn/Arg/Lys decarboxylase | 2.0 |
| AES_3004 putative amino acid permease | -5.5 |
| AES_3009 pilin biosynthetic protein | -1.8 |
| AES_3011 putative membrane protein | 2.3 |
| AES_3017 putative aminoglycoside phosphotransferase | 2.1 |
| AES_3025 conserved hypothetical protein | 8.8 |
| AES_3030 putative transcriptional regulator | 3.6 |
| AES_3048 conserved hypothetical protein | 10.4 |
| AES_3052 hypothetical protein | 5.5 |
| AES_3075 putative O-Methyltransferase | 10.2 |
| AES_3085 conserved hypothetical protein | 2.3 |
| AES_3094 putative secretion protein | 1.1 |
| AES_3098 putative transcriptional regulator | 2.1 |
| AES_3117 hypothetical protein | 2.4 |
| AES_3118 Probable phenazine biosynthesis protein | 2.8 |
| AES_3128 *phzF2* probable phenazine biosynthesis protein | -1.5 |
| AES_3135 probable sigma-70 factor, ECF subfamily | -5.3 |
| AES_3137 hypothetical protein | -5.5 |
| AES_3141 putative D-isomer specific 2-hydroxyacid dehydroge | -1.3 |
| AES_3143 putative transcriptional regulator, AraC family | -2.5 |
| AES_3178 Hypothetical protein | 19.4 |
| AES_3205 Hypothetical protein | 3.0 |
| AES_3223 putative transmembrane protein | -1.7 |
| AES_3245 *pqqB* pyrroloquinoline quinone biosynthesis protein B | 1.6 |
| AES_3264 *hmgA* homogentisate 1,2-dioxygenase | -7.2 |
| AES_3290 Resistance-Nodulation-Cell Division (RND) multidrug efflux membrane protein | -1.6 |
| AES_3305 putative methylase | 4.1 |
| AES_3328 putative binding protein component of ABC transporter | 2.5 |
| AES_3416 Hypothetical protein | 2.3 |
| AES_3465 Hypothetical protein | -1.5 |
| AES_3470 putative Zn-dependent alcohol dehydrogenase | -2.7 |
| AES_3495 hypothetical protein | -3.3 |
| AES_3496 putative signal transduction protein | 3.0 |
| AES_3502 *hcnC* hydrogen cyanide synthase | -1.7 |
| AES_3526 *pslF* possible glycosyl transferase | -2.5 |
| AES_3535 *pslN* putative DNA topoisomerase | -4.9 |
| AES_3545 *ansA* L-asparaginase I | -2.1 |
| AES_3569 conserved hypothetical protein | 12.7 |
| AES_3580 *arsR* arsenic resistance transcriptional regulator | -7.2 |
| AES_3632 putative flavin reductase dependent enzyme | -1.8 |
| AES_3636 putative sulfonate ABC transporter, periplasmic sulfonate-binding protein | 2.6 |
| AES_3637 putative ATP-binding component of ABC transporter | -2.1 |
| AES_3641 putative monooxygenase, DszC family | 1.7 |
| AES_3658 putative ABC transporter, periplasmic binding protein | -3.0 |
| AES_3715 hypothetical protein | -4.7 |
| AES_3733 putative ABC transporter, periplasmic substrate-binding protein | 3.0 |
| AES_3768 conserved hypothetical protein | 4.4 |
| AES_3781 hypothetical protein | 4.6 |
| AES_3793 hypothetical protein | 2.1 |
| AES_3795 hypothetical protein | -2.4 |
| AES_3851 putative deaminase | -2.4 |
| AES_3855 putative cyanate permease | -4.8 |
| AES_3870 *antA* anthranilate dioxygenase large subunit | 2.4 |
| AES_3872 *antC* anthranilate dioxygenase reductase | -3.0 |
| AES_3884 putative DNA-binding heavy metal response regulator | 1.5 |
| AES_3890 putative protease | 2.3 |
| AES_3893 putative protease | 2.9 |
| AES_3919 hypothetical protein | 2.1 |
| AES_3942 putative sulfate transporter | -2.0 |
| AES_3951 putative integrase | 2.4 |
| AES_3956 putative sensor/response regulator hybrid | -10.3 |
| AES_3966 putative transcriptional regulator, AraC family | 1.6 |
| AES_3993 *idh* monomeric isocitrate dehydrogenase | -3.5 |
| AES_4003 putative acyltransferase | -1.4 |
| AES_4006 putative isocitrate lyase | -2.4 |
| AES_4029 conserved hypothetical protein | 2.3 |
| AES_4033 putative permease | 4.1 |
| AES_4072 putative quinone oxidoreductase | 2.4 |
| AES_4092 conserved hypothetical protein | -2.0 |
| AES_4105 putative transcriptional regulator | 2.3 |
| AES_4146 hypothetical protein | 23.0 |
| AES_4152 conserved hypothetical protein | 3.2 |
| AES_4158 hypothetical protein | 3.7 |
| AES_4184 hypothetical protein | -4.0 |
| AES_4187 hypothetical protein | 2.6 |
| AES_4241 conserved hypothetical protein | -1.4 |
| AES_4244 putative RNA polymerase | 2.8 |
| AES_4247 hypothetical protein | 2.4 |
| AES_4249 hypothetical protein | -2.5 |
| AES_4257 putative ATPase | -2.8 |
| AES_4273 putative transcriptional regulator, LysR family | -1.6 |
| AES_4324 transglutaminase-like domain protein | -1.6 |
| AES_4334 putative two-component sensor histidine kinase | -4.0 |
| AES_4355 conserved hypothetical protein | 11.1 |
| AES_4372 putative integral membrane transport protein | 4.5 |
| AES_4374 putative ATP-binding component of ABC transporter | 1.8 |
| AES_4388 hypothetical protein | -5.3 |
| AES_4447 putative lipoprotein releasing system, ATP-binding protein | -1.4 |
| AES_4448 putative lipoprotein releasing system, permease protein | -2.8 |
| AES_4506 hypothetical protein | 9.4 |
| AES_4516 probable ATP-binding component of ABC taurine transporter | -1.4 |
| AES_4519 histone-like protein HU form N | -1.8 |
| AES_4529 putative D-alanyl-D-alanine carboxypeptidase | 2.2 |
| AES_4536 *pelB* conserved hypothetical protein | 5.6 |
| AES_4539 *pelE* hypothetical protein | 3.7 |
| AES_4560 putative von Willebrand factor type A domain | -3.8 |
| AES_4563 hypothetical protein | 2.3 |
| AES_4568 putative transcriptional regulator, AraC family | -2.9 |
| AES_4571 putative enoyl CoA-hydratase/isomerase family protein | -3.4 |
| AES_4591 conserved hypothetical protein | 1.9 |
| AES_4603 *metZ* o-succinylhomoserine sulfhydrylase | 1.4 |
| AES_4664 putative initiation factor 2 subunit | -4.5 |
| AES_4669 *ubiG* 3-demethylubiquinone-9 3-methyltransferase | 2.0 |
| AES_4697 putative two-component sensor | -1.3 |
| AES_4703 *yciB* putative intracellular septation protein | 5.3 |
| AES_4727 putative transcriptional regulator, AraC family | 1.7 |
| AES_4780 Putative recombination protein | -3.0 |
| AES_4805 conserved hypothetical protein | 3.5 |
| AES_4813 hypothetical protein | -2.9 |
| AES_4853 *plcN* non-hemolytic phospholipase C precursor | 3.2 |
| AES_4857 probable ABC transporter permease component | 19.7 |
| AES_4872 putative non-ribosomal peptide synthetase | 2.3 |
| AES_4887 putative sensor histidine kinase/response regulator | -2.1 |
| AES_4899 putative chemotaxis signal transduction protein | -3.1 |
| AES_4904 putative MFS transporter | 3.7 |
| AES_4922 *phnD* binding protein component of ABC phosphonate transporter | 4.7 |
| AES_4946 putative sigma-70 factor, ECF subfamily | 1.9 |
| AES_4948 conserved hypothetical protein | -1.4 |
| AES_4969 putative sulfonate binding protein | 1.3 |
| AES_5007 *rnfE* putative NADH:ubiquinone oxidoreductase subunit | 2.7 |
| AES_5011 *rnfB* putative NADH:ubiquinone oxidoreductase subunit | -4.7 |
| AES_5048 hypothetical protein | 1.8 |
| AES_5083 putative *recX* protein | -4.8 |
| AES_5132 *tsf* translation elongation factor | 7.6 |
| AES_5169 putative metal-transporting P-type ATPase | -1.8 |
| AES_5177 putative lipoprotein | 2.5 |
| AES_5186 *wspD* CheW domain protein | -2.6 |
| AES_5206 conserved hypothetical protein | 1.7 |
| AES_5207 putative transciptional regulator | 2.2 |
| AES_5256 putative N-acetylglucosamine-6-phosphate deacetylase | 8.6 |
| AES_5271 probable oxidoreductase | -1.7 |
| AES_5276 hypothetical protein | 24.1 |
| AES_5283 *guaB* inosine-5-monophosphate dehydrogenase | 3.0 |
| AES_5295 conserved hypothetical protein | -1.9 |
| AES_5322 *hscA* heat shock protein HscA | -2.6 |
| AES_5325 *ndk* nucleoside diphosphate kinase | -2.1 |
| AES_5380 putative long-chain-fatty-acid-CoA ligase | 1.9 |
| AES_5400 conserved hypothetical protein | 3.6 |
| AES_5439 conserved hypothetical protein | 3.9 |
| AES_5443 possible isochorismatase family protein | -1.9 |
| AES_5481 hypothetical protein | -2.6 |
| AES_5484  *sltB1* soluble lytic transglycosylase B | -1.8 |
| AES_5500 *hisD* histidinol dehydrogenase | -5.0 |
| AES_5503 conserved hypothetical protein | -1.3 |
| AES_5532 *phaF* polyhydroxyalkanoate synthesis protein | 6.0 |
| AES_5545 probable ABC transporter | 2.7 |
| AES_5554  *ribC* riboflavin synthase alpha chain | -2.0 |
| AES_5568 conserved hypothetical protein | 13.3 |
| AES_5633 putative efflux protein | 3.1 |
| AES_5687 putative ATP-binding component of ABC transporter | -2.2 |
| AES_5721  *rplW* 50S ribosomal protein L23 | -3.4 |
| AES_5777 Hypothetical protein | 8.2 |
| AES_5792 Hypothetical protein | 1.7 |
| AES_5800 conserved hypothetical protein | -3.0 |
| AES_5818 Hypothetical protein | 6.9 |
| AES_5820 conserved hypothetical protein | 4.0 |
| AES_5852 conserved hypothetical protein | -2.1 |
| AES_5925 *cafA* cytoplasmic axial filament protein | -2.0 |
| AES_5931 *mreC* rod shape-determining protein | 6.8 |
| AES_5950 putative binding protein component of ABC transporter | 2.9 |
| AES_5952 putative dipeptide ABC transport system permease | -1.5 |
| AES_6013 conserved hypothetical protein | 1.9 |
| AES_6022 *rtcR* transcriptional regulatory protein RtcR | 2.2 |
| AES_6056 putative nucleotide methyltransferase | 2.0 |
| AES_6057 conserved hypothetical protein | -1.3 |
| AES_6090 *prfA* peptide chain release factor 1 | 1.9 |
| AES_6111 hypothetical protein | 2.1 |
| AES_6116 conserved hypothetical protein | -1.4 |
| AES_6125 paraquat-inducible protein A-like protein | -1.5 |
| AES_6126 putative paraquat-inducible protein | 2.1 |
| AES_6147 putative sugar fermentation stimulation protein | 2.6 |
| AES_6167 conserved hypothetical protein | 1.2 |
| AES_6168 conserved hypothetical protein | 10.8 |
| AES_6197 Uncharacterized protein conserved in bacteria | -21.2 |
| AES_6214 putative methyltransferase | 1.5 |
| AES_6228 *selA* L-seryl-tRNA(ser) selenium transferase | 1.7 |
| AES_6278 *accC* biotin carboxylase | 3.3 |
| AES_6285 hypothetical protein | 7.5 |
| AES_6286 putative membrane protein, MarC family | 2.1 |
| AES_6290 putative permease of ABC transporter | -2.2 |
| AES_6317 putative transcriptional regulator | 3.8 |
| AES_6328 *ureF* urease accessory protein UreF | 3.3 |
| AES_6330 hypothetical protein | -2.1 |
| AES_6333 putative MFS transporter | 13.0 |
| AES_6364 putative membrane protein | 2.1 |
| AES_6381  *orn* oligoribonuclease | 9.4 |
| AES_6389 conserved hypothetical protein | 2.4 |
| AES_6423 hypothetical protein | 5.7 |
| AES_6489  *hemE* uroporphyrinogen decarboxylase | 16.0 |
| AES_6512 conserved hypothetical protein | -2.0 |
| AES_6529 putative poly(hydroxyalcanoate) granule-associated protein | 1.5 |
| AES_6550 *estA* esterase EstA | 1.7 |
| AES_6558 conserved hypothetical protein | 1.7 |
| AES_6562 *typA* GTP-binding protein TypA/BipA | -1.9 |
| AES_6583 putative ABC-type amino acid transporter | -1.8 |
| AES_6590 conserved hypothetical protein | -2.8 |
| AES_6600 putative short-chain alcohol dehydrogenase | -3.3 |
| AES_6654 *trxA* thioredoxin | -5.9 |
| AES_6657 putative acyltransferase | 4.2 |
| AES_6658 putative 3-octaprenyl-4-hydroxybenzoate carboxylyase | 2.3 |
| AES_6679 conserved hypothetical protein | -3.0 |
| AES_6709 putative hydrolase | 4.2 |
| AES_6726  *xpt* xanthine phosphoribosyltransferase | 16.9 |
| AES_6747 *algC* phosphomannomutase AlgC | -9.3 |
| AES_6792 *phoR* two-component sensor PhoR | 3.5 |
| AES_6800 putative lycine betaine/L-proline ABC transporter, ATP- binding | 1.6 |
| AES_6803 *cls* cardiolipin synthase | 7.0 |
| AES_6810 putative 3-hydroxyacyl-CoA dehydrogenase | 10.3 |
| AES_6820 hypothetical protein | 2.3 |
| AES_6838 *adhA* alcohol dehydrogenase, zinc-containing | 5.5 |
| AES_6875 *rmd* oxidoreductase Rmd | 3.0 |
| AES_6893 putative membrane protein | -2.1 |
| AES_6903 putative permease of ABC transporter | -6.7 |
| AES_6920 putative phosphatase | -1.9 |
| AES_6921 putative cyclopropan-fatty-acyl-phospholipid synthase | -6.7 |
| AES_6925 putative beta-lactamase | -1.9 |
| AES_6931 putative C4-type zinc finger protein, DksA/TraR family | 2.2 |
| AES_6932 putative cobalamin synthesis protein/P47K family protein | 1.3 |
| AES_6943 *atpA* ATP synthase alpha chain | -2.1 |
| AES_7005 hypothetical protein | 3.1 |
| AES_7033 hypothetical protein | -3.2 |
| AES_7036 hypothetical protein | 3.5 |
| AES_7070 hypothetical protein | -3.1 |
| AES_7082 hypothetical protein | -3.9 |
| AES_7105 hypothetical protein | 2.1 |
| AES_7111 hypothetical protein | -1.3 |
| AES_7122 hypothetical protein | 7.7 |
| AES_7127 hypothetical protein | -3.2 |
| AES_7136 hypothetical protein | 1.3 |
| AES_7137 hypothetical protein | -3.4 |
| AES_7140 hypothetical protein | 2.1 |
| AES_7165 hypothetical protein | 3.2 |
| AES_7167 conserved hypothetical protein | 8.4 |
| AES_7177 hypothetical protein | 3.5 |
| AES_7187 hypothetical protein | 6.1 |
| PA2G_00068 hypothetical protein | 2.1 |
| PA2G_00447 hypothetical protein | -2.6 |
| PA2G_00779 integrase | 2.7 |
| PA2G_00799 Cro-like prophage protein | 3.1 |
| PA2G_00800 hypothetical protein | 1.9 |
| PA2G_00803 hypothetical protein | 4.0 |
| PA2G_00873 predicted protein | -4.4 |
| PA2G_00970 hypothetical protein | 3.1 |
| PA2G_00996 hypothetical protein | 7.8 |
| PA2G_01018 hypothetical protein | 3.2 |
| PA2G_01041 hypothetical protein | -4.3 |
| PA2G_01228 predicted protein | 6.8 |
| PA2G_01527 hypothetical protein | -1.6 |
| PA2G_01851 hypothetical protein | -1.2 |
| PA2G_01862 hypothetical protein | 3.2 |
| PA2G_01866 hypothetical protein | -3.1 |
| PA2G_01996 dioxygenase large alpha subunit | -2.5 |
| PA2G_02008 hypothetical protein | 1.2 |
| PA2G_02035 hypothetical protein | 11.1 |
| PA2G_02036 hypothetical protein | 1.8 |
| PA2G_02039 putative betaine-aldehyde dehydrogenase | 4.2 |
| PA2G_02093 uridylate kinase *pyrH* | 6.4 |
| PA2G_02111 hypothetical protein | -1.8 |
| PA2G_02160 hypothetical protein | 3.9 |
| PA2G_02184 hypothetical protein | 5.4 |
| PA2G_02292 conserved hypothetical protein | -3.7 |
| PA2G_02334 RND multidrug efflux pump membrane fusion protein | 5.0 |
| PA2G_02340 hypothetical protein | -1.4 |
| PA2G_02932 hypothetical protein | 3.2 |
| PA2G_03173 conserved hypothetical protein | -2.9 |
| PA2G_05478 *pils2* pilin assembly protein | -4.3 |
| PA2G_05489 hypothetical protein | -3.1 |
| PA2G_05490 DNA topoisomerase | 3.3 |
| PA2G_05502 hypothetical protein | 2.5 |
| PA2G_05835 conserved hypothetical protein | -3.0 |
| PA2G_05976 predicted protein | 2.7 |
| PACG_00493 hypothetical protein | -1.2 |
| PACG_01378 Rhs family protein | -1.6 |
| PACG_04888 hypothetical protein | 2.6 |
| PACG_04897 hypothetical protein | 12.9 |
| PACG_04921 hypothetical protein | -4.1 |
| PA14_03285 conserved hypothetical protein | -4.5 |
| PA14_03350 hypothetical protein | 1.5 |
| PA14_12910 hypothetical protein | 2.0 |
| PA14_13200 conserved hypothetical protein | 3.1 |
| PA14_13970 hypothetical protein | 2.9 |
| PA14_20510 hypothetical protein | -5.2 |
| PA14_23360 O-antigen chain length terminator | -9.5 |
| PA14_23390 putative polysaccharide biosynthesis protein | -1.5 |
| PA14_30210 ATP-dependent Clp protease | -6.6 |
| PA14_30960 TraG-like protein | 5.8 |
| PA14_33980 hypothetical protein | -2.6 |
| PA14_35890 Putative aminotransferase | -3.2 |
| PA14_36790 hypothetical protein | 7.5 |
| PA14_38920 hypothetical protein | -2.1 |
| PA14_54880 putative serine acetyltransferase | -2.8 |
| PA14_54900 hypothetical protein | 1.4 |
| PA14_55020 possible ABC-type transporter, permease component | -7.4 |
| PA14_55040 putative ATP-binding protein | -3.5 |
| PA14_55090 hypothetical protein | -3.6 |
| PA14_58740 hypothetical protein | 5.4 |
| PA14_59190 hypothetical protein | -3.4 |
| PA14_59350 Type IV B pilus protein | 1.6 |
| PA14_59510 conserved hypothetical protein | -3.6 |
| PA14_59660 lytic murein transglycosylase | -2.6 |
| PA14_59720 putative pili assembly protein | 4.6 |
| PA14_59760 putative pili assembly protein | -6.6 |
| PA14_59910 conserved hypothetical protein | -3.8 |
| PA14_59980 hypothetical protein | 5.6 |
| PA14_60300 type 4 fimbrial biogenesis protein | 2.7 |
| PA14_61200 conserved_hypothetical protein | -1.5 |
| PA14_61410 conserved_hypothetical protein | 1.4 |
| PSPA7_0099 ultraviolet light resistance protein B | 2.8 |
| PSPA7_0679 hypothetical protein | -2.5 |
| PSPA7_0701 putative terminase endonuclease subunit | 2.6 |
| PSPA7_0926 cyclase family protein | -1.8 |
| PSPA7_1004 putative transcriptional regulator | 8.7 |
| PSPA7_1020 response regulator receiver modulated diguanylate phosphodiesterase | 12.9 |
| PSPA7_1219 hypothetical protein | 11.6 |
| PSPA7_1719 hypothetical protein | 2.9 |
| PSPA7_1843 hypothetical protein | -2.1 |
| PSPA7_2125 GntR family transcriptional regulator | 15.6 |
| PSPA7_2231 hypothetical protein | -1.7 |
| PSPA7_2281 transglutaminase family protein | 3.0 |
| PSPA7_2331 sensor histidine kinase/response regulator | 1.2 |
| PSPA7_2397 hypothetical protein | 2.2 |
| PSPA7_2424 anti-repressor protein Ant | -2.3 |
| PSPA7_2454 TPR repeat-containing protein | 1.4 |
| PSPA7_2464 hypothetical protein | 4.2 |
| PSPA7_2609 *vqsM* HTH-type transcriptional regulator | 5.6 |
| PSPA7_2621 *proQ* activator of osmoprotectant transporter ProP | 14.2 |
| PSPA7_2630 TetR family transcriptional regulator | 1.2 |
| PSPA7_2671 hypothetical protein | -2.0 |
| PSPA7_2685 putative acyl-CoA dehydrogenase | 1.3 |
| PSPA7_2791 putative filamentous haemagglutinin | 6.9 |
| PSPA7_3033 hypothetical protein | -2.4 |
| PSPA7_3034 hypothetical protein | 2.7 |
| PSPA7_3055 hypothetical protein | -2.0 |
| PSPA7_3114 hypothetical protein | 1.8 |
| PSPA7_3130 hypothetical protein | -1.5 |
| PSPA7_3153 glycogen branching enzyme | -3.1 |
| PSPA7_3216 hypothetical protein | 2.2 |
| PSPA7_3237 HAD family hydrolase | -4.8 |
| PSPA7_3271 SdiA-regulated protein | -4.0 |
| PSPA7_3311 cytochrome c550 | -3.4 |
| PSPA7_3315 hypothetical protein | 2.9 |
| PSPA7_3324 hypothetical protein | 2.1 |
| PSPA7_3366 hypothetical protein | 3.0 |
| PSPA7_3417 LasA protease precursor | -2.2 |
| PSPA7_3454 hypothetical protein | -2.8 |
| PSPA7_3620 hypothetical protein | 7.7 |
| PSPA7_3727 conjugal transfer protein | -2.0 |
| PSPA7_3823 hypothetical protein | -2.2 |
| PSPA7_3876 glycine rich protein | -9.2 |
| PSPA7_3887 Rhs element Vgr protein | 1.7 |
| PSPA7_4163 putative lipoprotein | -4.3 |
| PSPA7_4212 hypothetical protein | 3.3 |
| PSPA7_4277 *fliD* flagellar cap protein | -1.7 |
| PSPA7_4367 pimeloyl-CoA synthetase | 7.9 |
| PSPA7_4406 chaperone CupC2 | 1.8 |
| PSPA7_4422 hypothetical protein | -3.6 |
| PSPA7_4444 replicative DNA helicase | -3.1 |
| PSPA7_4453 putative DNA binding protein *amrZ* | 1.9 |
| PSPA7_4490 hypothetical protein | 5.8 |
| PSPA7_4655 hypothetical protein | 1.9 |
| PSPA7_4673 hypothetical protein | -4.0 |
| PSPA7_4799 hypothetical protein | -1.9 |
| PSPA7_4834 transmembrane sensor | -1.6 |
| PSPA7_5066 hypothetical protein | 1.4 |
| PSPA7_5067 putative tail tape measure protein | -5.1 |
| PSPA7_5074 nucleoid DNA-binding protein | -5.8 |
| PSPA7_5124 hypothetical protein | 3.5 |
| PSPA7_5130 hypothetical protein | 2.2 |
| PSPA7_5222 hypothetical protein | 4.4 |
| PSPA7_5262 hypothetical protein | -2.1 |
| PSPA7_5342 integrase/recombinase | 3.0 |
| PSPA7_5357 [AlpA family transcriptional regulator](http://rna.med.monash.edu.au/wasabi/view.cgi?who=ZZ;stop=5524330;chr=1;gid=Pa_PA7;start=5524542) | 3.1 |
| PSPA7_5398 PqiA family protein | -5.5 |
| PSPA7_5411 hypothetical protein | 22.4 |
| PSPA7_5459 cytidine deaminase | 4.7 |
| PSPA7_5881 hypothetical protein | 5.6 |
| PSPA7_6042 methyl-accepting chemotaxis protein | 2.8 |
| PSPA7_6052 transcriptional factor | -2.8 |
| PSPA7_6054 hypothetical protein | -1.4 |
| PSPA7_6061 Site-specific phage integrase family protein | -2.6 |
| PSPA7_6104 probable cytochrome c(mono-heme type) | 1.9 |
| PSPA7_6137 hypothetical protein | 1.8 |
| PSPA7_6249 short-chain dehydrogenase | 8.5 |
| PA7_5977 * hypothetical protein | 13.1 |
| PA7_6285 * hypothetical protein | 4.7 |
| PaerPA_01000833 hypothetical protein | -5.1 |
| PaerPA_01000873 hypothetical protein | 3.8 |
| PaerPA_01000876 single-stranded DNA binding protein | -1.5 |
| PaerPA_01000885 hypothetical protein | 5.1 |
| PaerPA_01000933 probable permease of ABC transporter | -1.9 |
| PaerPA_01001563 probable ring-hydroxylating dioxygenase subunit | -2.0 |
| PaerPA_01002922 hypothetical protein | 1.3 |
| PaerPA_01003055 hypothetical protein | -2.9 |
| PaerPA_01003082 hypothetical protein | 4.3 |
| PaerPA_01003096 conserved hypothetical protein | 1.4 |
| PaerPA_01003098 hypothetical protein | -4.2 |
| PaerPA_01003112 hypothetical protein | 2.6 |
| PaerPA_01003121 hypothetical protein | 1.9 |
| PaerPA_01003136 hypothetical protein | -2.4 |
| PaerPA_01003146 Uncharacterised membrane-anchored protein conserved in bacteria | 5.1 |
| PaerPA_01003149 hypothetical protein | 6.0 |
| PaerPA_01003151 hypothetical protein | 2.1 |
| PaerPA_01003221 hypothetical protein | 4.2 |
| PaerPA_01003304 conserved hypothetical protein | -1.4 |
| PaerPA_01003305 hypothetical protein | -5.8 |
| PaerPA_01003307 putative ATP/GTP-binding protein | 2.1 |
| PLES_07181 *phzM* putative phenazine-specific methyltransferase | -1.4 |
| PLES_12831 hypothetical protein | -2.3 |
| PLES_13231 hypothetical protein | 1.5 |
| PLES_13401 hypothetical protein | -8.0 |
| PLES_13431 hypothetical protein | 5.2 |
| PLES_13521 putative portal protein | -1.8 |
| PLES_13531 putative Clp protease | -2.4 |
| PLES_13551 hypothetical protein | 2.9 |
| PLES_13591 hypothetical protein | 2.1 |
| PLES_13631 hypothetical protein | 3.3 |
| PLES_23471 hypothetical protein | 4.8 |
| PLES_23561 Superfamily I DNA and RNA helicases | 2.0 |
| PLES_25171 cI repressor protein | 6.4 |
| PLES_25211 hypothetical protein | -1.3 |
| PLES_25391 hypothetical protein | -1.4 |
| PLES_25401 portal protein | 2.2 |
| PLES_25531 hypothetical protein | -2.8 |
| PLES_25591 hypothetical protein | -5.9 |
| PLES_25651 hypothetical protein | 2.7 |
| PLES_25922 *pltK* inner membrane permease protein | -4.6 |
| PLES_26101 heavy metal transport/detoxification protein | -3.6 |
| PLES_26261 heavy metal translocating P-type ATPase | 3.8 |
| PLES_26961 hypothetical protein | 3.1 |
| PLES_29101 *pvdP* periplasmic pyoverdine protein | -1.6 |
| PLES_31031 putative glucose sensitive porin | -2.3 |
| Pae_0010** hypothetical protein | -3.9 |
| Pae_0219** probable transcriptional regulator | 3.6 |
| Pae_0838** hypothetical protein | -5.0 |
| Pae_2715** hypothetical protein | -1.6 |
| Pae_2718** hypothetical protein | -1.5 |
| Pae_2815** hypothetical protein | -2.2 |
| Pae_3572** hypothetical protein | -2.2 |
| Pae_4118** hypothetical protein | 5.0 |
| Pae_4124** hypothetical protein | 2.2 |
| Pae_4157** hypothetical protein | 1.8 |
| Pae_4210** conserved hypothetical protein | -3.1 |
| PA0003 *recF* DNA replication and repair protein | -2.6 |
| PA0215 probable transporter | 8.3 |
| PA0216 probable transporter | -2.5 |
| PA0608 probable phosphoglycolate phosphatase | 4.8 |
| PA0783 *putP* sodium/proline symporter | 1.7 |
| PA0998 putative beta-keto-acyl-acyl-carrier protein synthase | -5.2 |
| PA1099 two-component response regulator | 3.9 |
| PA1108 probable major facilitator superfamily (MFS) protein | 1.7 |
| PA1109 probable transcriptional regulator | -2.8 |
| PA1381 hypothetical protein | -2.2 |
| PA1394 hypothetical protein | 2.6 |
| PA1396 probable two-component response regulator | -3.1 |
| PA1399 probable transcriptional regulator | 9.3 |
| PA1400 probable pyruvate carboxylase | 4.8 |
| PA1640 conserved hypothetical protein | 3.9 |
| PA1727 conserved hypothetical protein | 12.6 |
| PA1728 hypothetical protein | 3.7 |
| PA2406 hypothetical protein | 2.7 |
| PA2411 probable thioesterase | -3.0 |
| PA2714 probable molybdopterin oxidoreductase | 3.0 |
| PA2746 hypothetical protein | 1.5 |
| PA2772 hypothetical protein | 3.1 |
| PA2776 conserved hypothetical protein | -1.5 |
| PA2831 conserved hypothetical protein | 3.3 |
| PA3028 molybdenum cofactor biosynthesis protein A2 | -1.8 |
| PA3136 probable secretion protein | 3.2 |
| PA3380 conserved hypothetical protein | 2.4 |
| PA3501 hypothetical protein | -2.6 |
| PA3511 probable short-chain dehydrogenase | 1.7 |
| PA3517 probable lyase | -3.5 |
| PA3527 *pyrC* dihydroorotase | 12.1 |
| PA4241 30S ribosomal protein S13 | -9.5 |
| PA4429 probable cytochrome c1 precursor | -4.3 |
| PA4896 probable sigma-70 factor, ECF subfamily | 2.5 |

§ Fold change indicates up or down regulated in AES-1M.

Gene ID codes: PaerPA = *P. aeruginosa* PACS2, PA14 = *P. aeruginosa* UCBPP-PA14,

PSPA7 and PA7= *P. aeruginosa* PA7, PA2G = *P. aeruginosa* 2192, PACG = *P. aeruginosa* c3719,

PLES and Pae = *P. aeruginosa* LESB58. *Gene identified in PA7 but not in PSPA7 **Gene identified in Pae but not PLES.
