# Supplementary material for: Pseudomonas aeruginosa AES-1 Exhibits Increased Virulence Gene Expression during Chronic Infection of Cystic Fibrosis Lung
Source: PLoS One. 2011 Sep 15;6(9):e24526. doi: 10.1371/journal.pone.0024526 (PMC3174184; doi:10.1371/journal.pone.0024526)
Supplement: Table S3 — Genes without homologues in PAO1* that were differentially expressed between P. aeruginosa AES-1R and AES-1M grown in ASMDM (p<0.05). (DOC) [file pone.0024526.s003.doc]

**Supplementary Table 2: Genes without homologues in PAO1* that were differentially expressed between *P. aeruginosa* AES-1R and AES-1M grown in ASMDM (p<0.05).**

| **Gene ID** | **Description** | **p-value** | **Fold-Change§** |
| --- | --- | --- | --- |
| PaerPA_01000873 | Pathogenesis-related protein PAGI-5 | 0.00009 | **3.8** |
| PA14_59660 | Hypothetical protein | 0.00113 | **-2.6** |
| PSPA7_4163 | Polyketide synthase | 0.00215 | **-4.3** |
| PA14_23390 | Putative polysaccharide biosynthesis protein | 0.00249 | **-1.5** |
| PSPA7_0679 | Hypothetical protein | 0.00303 | **-2.5** |
| PLES_13281 | Hypothetical protein | 0.00497 | **-1.5** |
| PLES_23731 | Hypothetical protein | 0.00515 | **1.8** |
| PLES_13431 | Hypothetical protein | 0.00548 | **5.2** |
| PLES_13521 | Putative portal protein | 0.00570 | **-1.8** |
| PSPA7_0701 | Phage terminase subunit | 0.00737 | **2.6** |
| PSPA7_6054 | GTPase (dynamin-related) | 0.00808 | **-1.4** |
| PA2G_05976 | Predicted protein | 0.00811 | **2.7** |
| PLES_23471 | Hypothetical protein | 0.00819 | **4.8** |
| PA14_59910 | Hypothetical protein | 0.00917 | **-3.8** |
| PA14_59190 | Luminal binding protein | 0.00936 | **-3.4** |
| PLES_25391 | Probable signal peptide | 0.01067 | **-1.4** |
| PSPA7_5357 | Phage transcriptional protein | 0.01095 | **3.1** |
| PSPA7_0099 | Ultraviolet light resistance protein B | 0.01108 | **2.8** |
| PLES_25211 | Hypothetical protein | 0.01195 | **-1.3** |
| PSPA7_4490 | Pathogenesis-related protein PAGI-5 | 0.01204 | **5.8** |
| PaerPA_01003136 | Membrane protein | 0.01289 | **-2.4** |
| PLES_25591 | Hypothetical protein | 0.01305 | **-5.9** |
| PA2G_02184 | Phage-related integrase | 0.01463 | **5.4** |
| PLES_26961 | Conserved hypothetical protein | 0.01519 | **3.1** |
| PA2G_02093 | Uridylate kinase *pyrH* | 0.01667 | **6.4** |
| PLES_13301 | Hypothetical protein | 0.01705 | **-1.6** |
| PA2G_01018 | Hypothetical protein | 0.01728 | **3.2** |
| PaerPA_01003098 | ABC-type amino acid transport signal transduction periplasmic component domain | 0.01746 | **-4.2** |
| PSPA7_6052 | Transcriptional factor | 0.01927 | **-2.8** |
| PA14_59980 | Secreted protein | 0.02299 | **5.6** |
| PA2G_00803 | Bacteriophage lambda protein | 0.02388 | **4.0** |
| PA2G_00779 | Integrase family protein | 0.02431 | **2.7** |
| PaerPA_01000833 | Hypothetical protein | 0.02449 | **-5.1** |
| PA2G_01851 | Hypothetical protein | 0.02459 | **-1.2** |
| PLES_25531 | Phage protein | 0.02728 | **-2.8** |
| PLES_08061 | Phage antitermination protein Q | 0.02820 | **-1.4** |
| PaerPA_01003307 | Phage plasmid-related protein tigr03299 | 0.03052 | **2.1** |
| PLES_25651 | Hypothetical protein | 0.03093 | **2.7** |
| PACG_04921 | Hypothetical protein | 0.03107 | **-4.1** |
| PACG_00493 | Hypothetical protein | 0.03194 | **-1.2** |
| PA2G_01527 | Crispr-associated helicase cas3 family | 0.03222 | **-1.6** |
| PaerPA_01003112 | Secreted protein | 0.03246 | **2.6** |
| PSPA7_3216 | Hypothetical protein | 0.03285 | **2.2** |
| PA2G_00996 | Phage integrase | 0.03308 | **7.8** |
| PLES_13591 | Hypothetical protein | 0.03309 | **2.1** |
| PA14_03285 | Cytoplasmic protein | 0.03345 | **-4.5** |
| PLES_13551 | Hypothetical protein | 0.03353 | **2.9** |
| PA2G_05835 | Conserved hypothetical protein | 0.03445 | **-3.0** |
| PSPA7_6285 | Hypothetical protein | 0.03620 | **4.7** |
| PA2G_00970 | Hypothetical protein | 0.03622 | **3.1** |
| PLES_25401 | Phage portal protein hk97 family | 0.03623 | **2.2** |
| PSPA7_2424 | Ant protein | 0.03634 | **-2.3** |
| PLES_13631 | Hypothetical protein | 0.03635 | **3.3** |
| PLES_13231 | Hypothetical protein | 0.03667 | **1.5** |
| PA14_03350 | Keratin-associated protein 4 family | 0.03739 | **1.5** |
| PaerPA_01003151 | Transposase is4 family protein | 0.03850 | **2.1** |
| PA2G_00800 | Hypothetical protein | 0.03873 | **1.9** |
| PA14_59510 | Hypothetical protein | 0.04061 | **-3.6** |
| PA14_58740 | Hypothetical protein | 0.04113 | **5.4** |
| PLES_13401 | Replication protein A C-terminal | 0.04130 | **-8.0** |
| PSPA7_0926 | Cyclase family protein | 0.04250 | **-1.8** |
| PA14_54900 | Saccharopine dehydrogenase | 0.04295 | **1.4** |
| PSPA7_3237 | Outer membrane efflux protein | 0.04321 | **-4.8** |
| PaerPA_01003096 | Hypothetical protein | 0.04392 | **1.4** |
| PaerPA_01000885 | Hypothetical protein | 0.04407 | **5.1** |
| PaerPA_01003121 | Secreted protein | 0.04436 | **1.9** |
| PSPA7_6061 | Site-specific phage integrase family protein | 0.04484 | **-2.6** |
| PA2G_02111 | Hypothetical protein | 0.04567 | **-1.8** |
| PA14_59350 | Shufflon proteinA type IV pilus protein | 0.04580 | **1.6** |
| PLES_23561 | Superfamily I: DNA and RNA helicases | 0.04752 | **2.0** |
| PA2G_05502 | Hypothetical protein | 0.04977 | **2.5** |
| PA2G_00799 | Conserved domain protein | 0.04987 | **3.1** |
| PaerPA_01003149 | Gnat family | 0.01047 | **6.0** |
| PaerPA_01003146 | Membrane-anchored protein | 0.01679 | **5.1** |

*E-value less than 10^-4^.

§ Fold-change indicates up or downregulated in AES-1M.

Gene ID codes: PaerPA = *P. aeruginosa* PACS2, PA14 = *P. aeruginosa* UCBPP-PA14,

PSPA7 = *P. aeruginosa* PA7, PA2G = *P. aeruginosa* 2192, PACG = *P. aeruginosa* c3719,

PLES = *P. aeruginosa* LESB58.
